# Loss of MIR503HG facilitates papillary renal cell carcinoma associated lymphatic metastasis by triggering NOTCH1/VEGFC signaling

**DOI:** 10.7150/ijbs.83302

**Published:** 2023-06-19

**Authors:** Yiqiu Wang, Xinyi Zheng, Wenjie Huang, Jiayi Lu, Naiqiao Hou, Jiabao Qi, Junjie Ma, Wei Xue, Junhua Zheng, Wei Zhai

**Affiliations:** 1 Department of Urology, Renji Hospital, Shanghai Jiao Tong University School of Medicine, Shanghai, 200127, China.; 2 Department of Pharmacy, Huashan Hospital, Fudan University, 12 Middle Urumqi Road, Shanghai, 200040, China.; 3 Department of Urology, Yangpu Hospital, School of Medicine, Tongji University, Shanghai, 200090, China.; 4 State Key Laboratory of Oncogenes and Related Genes, Department of Urology, Renji Hospital, School of Medicine, Shanghai Jiao Tong University, Shanghai, 200127, China.

**Keywords:** Papillary renal cell carcinoma, Lymphatic metastasis, Long non-coding RNA, DNA methylation, Target therapy

## Abstract

Clinical lymphatic metastasis indicates an extremely poor prognosis. Patients with papillary renal cell carcinoma (pRCC) have a high probability of progressing to lymphatic metastasis. However, the molecular mechanism of pRCC-associated lymphatic metastasis has not been elucidated. In this study, we found a downregulated long non-coding RNA (lncRNA) MIR503HG in pRCC primary tumor tissues due to hypermethylation at the CpG islands within its transcriptional start site. Decreased MIR503HG expression could stimulate tube formation and migration of human lymphatic endothelial cell (HLEC) and play a central role to promote lymphatic metastasis in vivo by enhancing tumor lymphangiogenesis. MIR503HG, located in the nucleus, bound with histone variant H2A.Z and affected the recruitment of histone variant H2A.Z to chromatin. Subsequently, increasing the H3K27 trimethylation caused by MIR503HG-overexpression epigenetically downregulated the NOTCH1 expression, which ultimately resulted in decreasing VEGFC secretion and lymphangiogenesis. Additionally, downregulated MIR503HG facilitated the HNRNPC expression, which ultimately promoted the maturation of NOTCH1 mRNA. Notably, upregulating MIR503HG expression might decrease pRCC resistance to the mTOR inhibitor. Together, these findings highlighted a VEGFC-independent mechanism of MIR503HG-mediated lymphatic metastasis. MIR503HG, identified as a novel pRCC-suppressor, would serve as the potentially biomarker for lymphatic metastasis.

## Introduction

Papillary renal cell carcinoma (pRCC), which varies widely from other RCC histologic variants, has its genetic and pathologic characteristics 1. Ranked as the second-most type of RCC, it represents 10-15% of all kidney cancers 2, 3. Different clinical behaviors were discovered in RCC subtypes because of biological distinction 4, 5. As the pattern of manifestation of biological underpinnings, pRCC has greater tendency to become metastatic pRCC (mpRCC) and spreads in different sites in patients. Compared with other subtypes, lymph node (LN) is recognized as the most common metastatic site in pRCC 6. Up to now, this unique biologic profile of pRCC has not been elucidated.

Through the lymphatic system, cancer cells could be disseminated to lymph-vessels and transported to distant LNs, finally settled down and colonized in LN 7. Therefore, lymphangiogenesis, as an essential process for lymphatic metastasis, was associated with metastasis-free survival in a dozen solid tumors 8, 9.

Previous studies reported the molecular markers can distinguish lymphatic vessels from blood vessels. Vascular endothelial growth factor C, named VEGFC, could induce lymphangiogenesis in tumor tissues 10. Given that the VEGFC/VEGF receptor 3 signaling was the key pathway for lymphatic metastasis 11, 12, multiple targeted drugs (anti-lymphangiogenic) have undergone clinical trials, such as monoclonal antibodies VGX-100 and IMC-3C5 13, 14. Understanding the specific mechanisms of VEGFC signaling pathway in pRCC would provide valuable treatment options for effective anti-VEGFC treatment in the clinic.

Long non-coding RNA (lncRNA) is a type of transcripts that are longer than two hundred nucleotides 15. Although exhibiting no protein-coding potential, it was reported that it involved in many biological processes, including regulating chromatin looping and modification 16, functioning as enhancer-associated RNAs 17, 18, trapping for some transcription factors 19, etc. Recent years, several researches have gradually unveiled the pathophysiological contributions of lncRNAs in the lymphatic metastasis 20-22. Untill now, few studies have explored the function and mechanism of lncRNAs of pRCC-associated lymphatic metastasis.

In the present study, we discovered MIR503HG, a downregulated lncRNA in pRCC, exerted a pivotal role in the VEGFC axis. Our finding revealed a novel mechanism of MIR503HG-related lymphatic metastasis for pRCC.

## Results

### The pRCC-associated lncRNA-MIR503HG negatively correlated with VEGFC secretion

**Figure 1:**
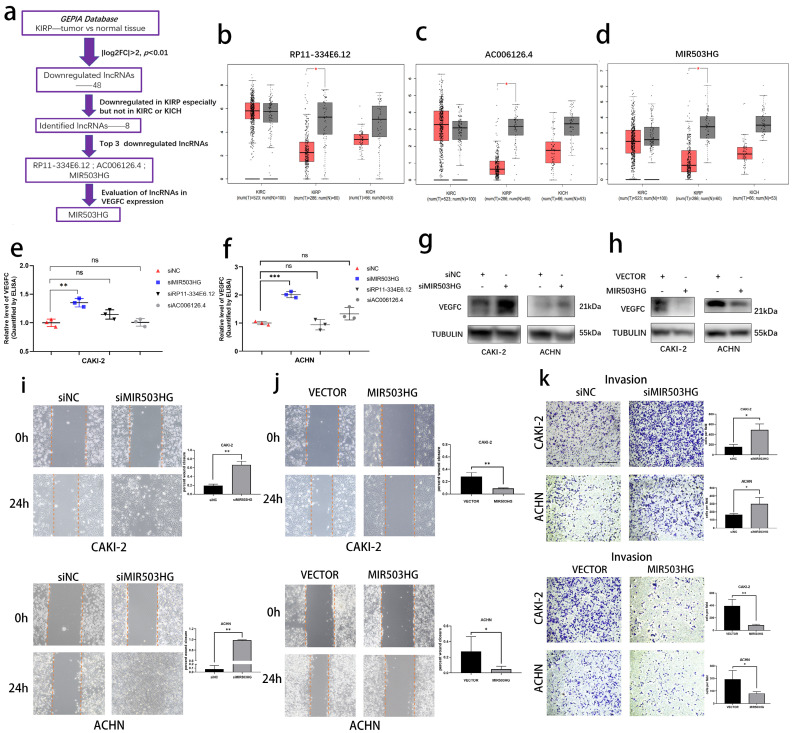
** The pRCC-associated lncRNA-MIR503HG negatively correlated with VEGFC secretion.** (a) Flow chart for the identification of VEGFC-associated lncRNA MIR503HG from GEPIA datasets. b-d. Downregulated of Non-coding RNA RP11-334E6.12 (b), AC006126.4 (c) MIR503HG (d) were found in TCGA-KIRP dataset. e-f. ELISA: After three LncRNA knockdown, the levels of VEGFC secretion were measured. Condition media were derived from CAKI-2 (e) and ACHN (f) cells, respectively. g-h. Representative image of Western blotting analysis of VEGFC protein expression in MIR503HG depletion (g) and overexpression (h) in pRCC cells. i-j. Representative images of the wound-healing assay using pRCC cell lines showing cell motility after ectopic expression (j) or knockdown of MIR503HG (i) at indicated time are shown. k. Invasion assays of pRCC cells with MIR503HG knockdown and overexpression. In all panels, data are shown as the mean ± s.d. For all representative images, at least three independent experiments were performed with similar results. NS, not significant, *P < 0.05, **P < 0.01, ***P < 0.001, one-way ANOVA followed by Dunnett's post hoc test was performed in e-f. The two-tailed Student's t-test was used in i-k, and the nonparametric Mann-Whitney U test was used in b-d.

Using transcriptome data from Gene Expression Profiling Interactive Analysis (GEPIA) database, we determined lymphatic metastasis-related lncRNAs in pRCC (**Fig. 1a**). In short, we firstly selected 48 eligible lncRNAs (**Table 1**) and then 8 lncRNAs were identified as the pRCC-associated lncRNAs, all of them were only downregulated in kidney renal papillary carcinoma (KIRP), but not in kidney renal clear cell carcinoma (KIRC) or kidney chromophobe (KICH). Top 3 downregulated lncRNAs were selected as candidates to explore the crucial lncRNA involved in pRCC-associated lymphatic metastasis (**Fig. 1b-d**). We respectively established lncRNA-knockdown pRCC cell lines: CAKI-2 and ACHN. These two cell lines were widely reported the harbor genomic characteristics of pRCC 25, 26. Next, with the ELISA assays, we found that MIR503HG was negatively associated with the VEGFC secretion in both CAKI-2 and ACHN cells (**Fig. 1e-f**), which was then verified by western blotting assay (**Fig. 1g-h**). Shifting the emphasis from three candidates to LncRNA-MIR503HG, we next established stable MIR503HG knockdown (shRNA) and overexpression pRCC cell lines for further research. The efficiency of siRNA, shRNA and overexpression were confirmed by qRT-PCR (**Supplemental Fig. 1a-h**). MIR503HG, as a tumor suppressor gene, could inhibit several solid tumors' capacity of progression 23, 24. Our results showed that MIR503HG knockdown significantly enhanced invasion (Transwell assays) and migration (Wound-healing assays) of CAKI-2 and ACHN cells compared with the control group. Correspondingly, compared with negative control group, MIR503HG overexpression reduced not only migratory but also invasive capacity in both CAKI-2 and ACHN cells (**Fig. 1i-k**).

### Downregulated MIR503HG related with poor prognosis and lymphatic metastasis of pRCC

**Figure 2:**
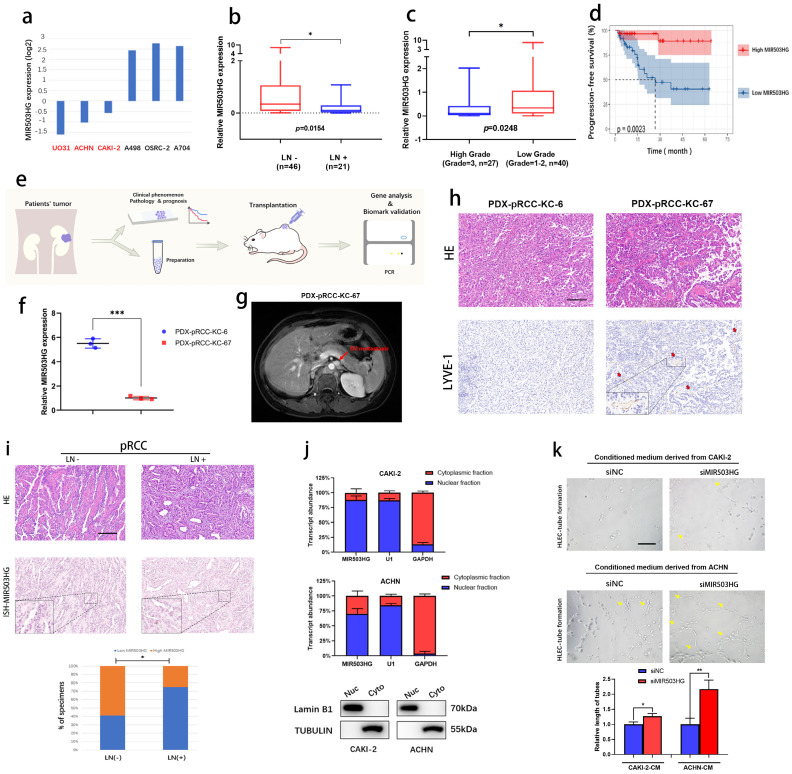
** Downregulated MIR503HG related with poor prognosis and lymphatic metastasis of pRCC.** a. CCLE database (the Cancer Cell Line Encyclopedia): lower expression of lncRNA MIR503HG were found in pRCC cell lines (ACHN, CAKI-2 and UO-31). b-c. Comparison of MIR503HG expression in LN-positive & LN-negative pRCC tissues (b) and high grade & low grade pRCC tissues (c). d. Kaplan-Meier survival analysis of PFS for pRCC patients with low versus high MIR503HG expression. Median expression was used as a cutoff value. e. Flow chart: PDX models were established for subsequent research. f. qRT-PCR analysis verified the expression level of MIR503HG between patient KC6 and patient KC67. g. Magnetic Resonance Imaging (MRI) showed the para-aortic nodes metastases in pRCC Patient (ID: KC67). h. Representive images of HE&IHC staining (LYVE-1) evaluating potential microlymphatic metastases in PDXs tumor tissue. i. Representative images of MIR503HG expression in LN-negative and LN-positive pRCC tissues, MIR503HG expression level were quantified by ISH. Scale bars: 100 μm. j. Subcellular fractionation analysis of MIR503HG subcellular allocation in CAKI-2 and ACHN cells (bottom: western blot; top: qRT-PCR). U1 snRNA (nuclear retained) and GAPDH mRNA (exported to cytoplasm) were used as controls. k. Representative images and quantification of tube formation for HLECs treated with culture media from CAKI-2/ACHN siNC and siMIR503HG cells. Scale bars: 50 μm. The nonparametric Mann-Whitney U test was used to compare the expression levels of the 2 groups in b and c, and 2-tailed Student's t-test in f and k; and the χ2 test in i and j. Error bars show the standard deviation (SD) from at least three independent experiments. *P < 0.05; **P < 0.01, ***P < 0.001.

As expected, we found that MIR503HG expression was especially downregulated in several pRCC cell lines (UO31, ACHN and CAKI-2) in the Cancer Cell Line Encyclopedia (CCLE) datasets (**Fig. 2a**). Based on the qRT-PCR results of Renji pRCC-cohort, we observed that lower expression of MIR503HG was associated with high rate of lymphatic metastasis (**Fig. 2b**), high tumor grade (**Fig. 2c**) and poor PFS (**Fig. 2d**). Using the pRCC tissues from patients, we established patient derived xenograft (PDX) models for determining its suppressive role in pRCC-associated lymphatic metastasis (**Fig. 2e**). Patient-KC67 with the lower MIR503HG expression was diagnosed as the lymphatic metastasis, which was examined by magnetic resonance imaging (MRI) during follow-up (**Fig. 2f-g**). Patient-KC67 also showed high microlymphatic vessel density, which was indicated by LYVE-1 positive region (**Fig. 2h**). Two patients' clinical information were collected (**Table 2**).

ISH assays were also performed by using formalin fixed paraffin-embedded (FFPE) tissues obtained from pRCC patients. Statistical analysis revealed that MIR503HG expression was significantly associated with lymphatic metastasis, suggesting it might be a prognostic factor of pRCC **(Fig. 2i)**. As expected, we noticed that MIR503HG could be detected in pRCC cells' nucleus in subcellular fractionation assay **(Fig. 2j)**, which was consistent with results of ISH.

Furthermore, tube formation experiments were performed in order to explore regulatory function of MIR503HG in lymphangiogenesis of pRCC. HLECs-tube formation was significantly inhibited after incubating with conditioned medium from MIR503HG overexpression pRCC cells (**Supplemental Fig. 2a**), while promoted in the siMIR503HG pRCC cells, compared with the control group, respectively (**Fig. 2k**).

### MIR503HG was downregulated in pRCC due to hypermethylation

**Figure 3:**
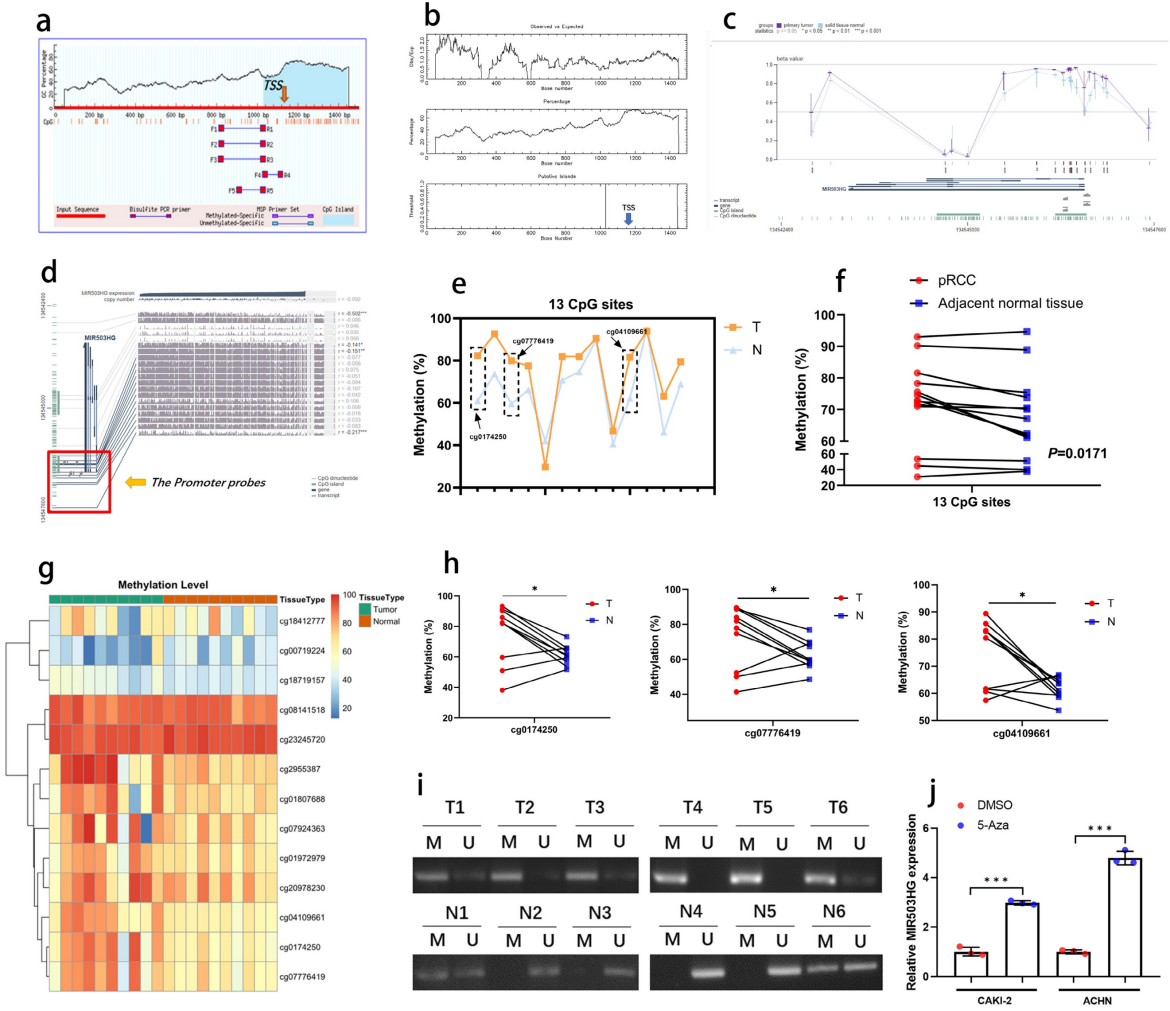
** MIR503HG was downregulated in pRCC due to hypermethylation.** a. Detection of CpG islands in MIR503HG promoter by MethPrimer. The horizontal axis of the curved lines represents the sequence of MIR503HG, and the vertical axis of the curved lines represents GC percentage. TSS: Transcription Start Sites. b. Detection of CpG islands in MIR503HG promoter by EMBOSS. TSS: Transcription Start Sites. c. The relative methylation level of primary tumor was higher than normal tissue (shown by MEXPRESS). d. 13 CpG sites of MIR503HG promoter region in TCGA-KIRP data (shown by MEXPRESS). e. The DNA methylation level of MIR503HG promoter was detected in 10 paired pRCC tissues and adjacent non-cancer tissues by bisulfite sequencing. f. Representative sequencing analysis from clinical samples revealed that methylated CpG sites in pRCC tissues were higher than the adjacent normal renal tissues. g. Heatmap: Methylation level of the 13 CpG sites in pRCC and paired normal tissue. h. Representative sequencing analysis: the methylation level of 3 CpG sites were significant higher in pRCC samples. i. MSP-PCR: Comparison of the DNA methylation level of MIR503HG promoter region in pRCC and paired normal renal tissues. M = Methylated, U = Unmethylated. j. Treatment with 5-aza-dC increased MIR503HG expression by demethylating promoter region of MIR503HG in pRCC cells. M = Methylated, U = Unmethylated. The Wilcoxon Signed rank test were used in c,e, f and h. The statistical difference was assessed through 2-tailed Student's t- test in j. *P < 0.05; **P < 0.01.

In consideration of DNA methylation is the dominating mechanism regulating lncRNA expression, bioinformatic data mining was conducted, and DNA hypermethylation status was observed at the transcription start site of MIR503HG in both MethPrimer and EMBOSS database (**Fig. 3a-b**). As the data from MEXPRESS database shown that methylation level was higher in primary pRCC than normal tissue (**Fig. 3c**), there may be a negative correlation between methylation status and MIR503HG expression.

We proposed that the downregulation of MIR503HG in tumor tissues was caused by DNA hypermethylation, a mainly epigenetic mechanism for eukaryotic genomes. Furthermore, refer to MEXPRESS, we selected 13 CpGs at MIR503HG promoter (**Fig. 3d**) to explore the DNA methylation status (cg-IDs were presented in** Table 3**). By bisulfite analysis, the methylation level of 13 CpGs was measured in 10 pairs of pRCC tumors and adjacent normal tissues. The average methylation status of CpG sites was higher in pRCC tissue than that in paired normal renal tissue (**Fig. 3e-g**). The methylation level of 3 specific CpGs (cg0174250, cg07776419, cg04109661) was remarkably increased in pRCC samples, but no significant difference was found in other CpGs (**Fig. 3h, Supplemental Fig. 3a-j**). MSP-PCR assays also confirmed that MIR503HG was significantly hypermethylated in pRCC tissues than in paired normal tissue (**Fig. 3i**). Importantly, the expression of MIR503HG in pRCC cells were induced after treated with 5'azacytidine (**Fig. 3j**). These results supported the hypothesis that DNA hypermethylation played a crucial role in the decrease of MIR503HG in pRCC.

### MIR503HG directly bound with H2A.Z

**Figure 4:**
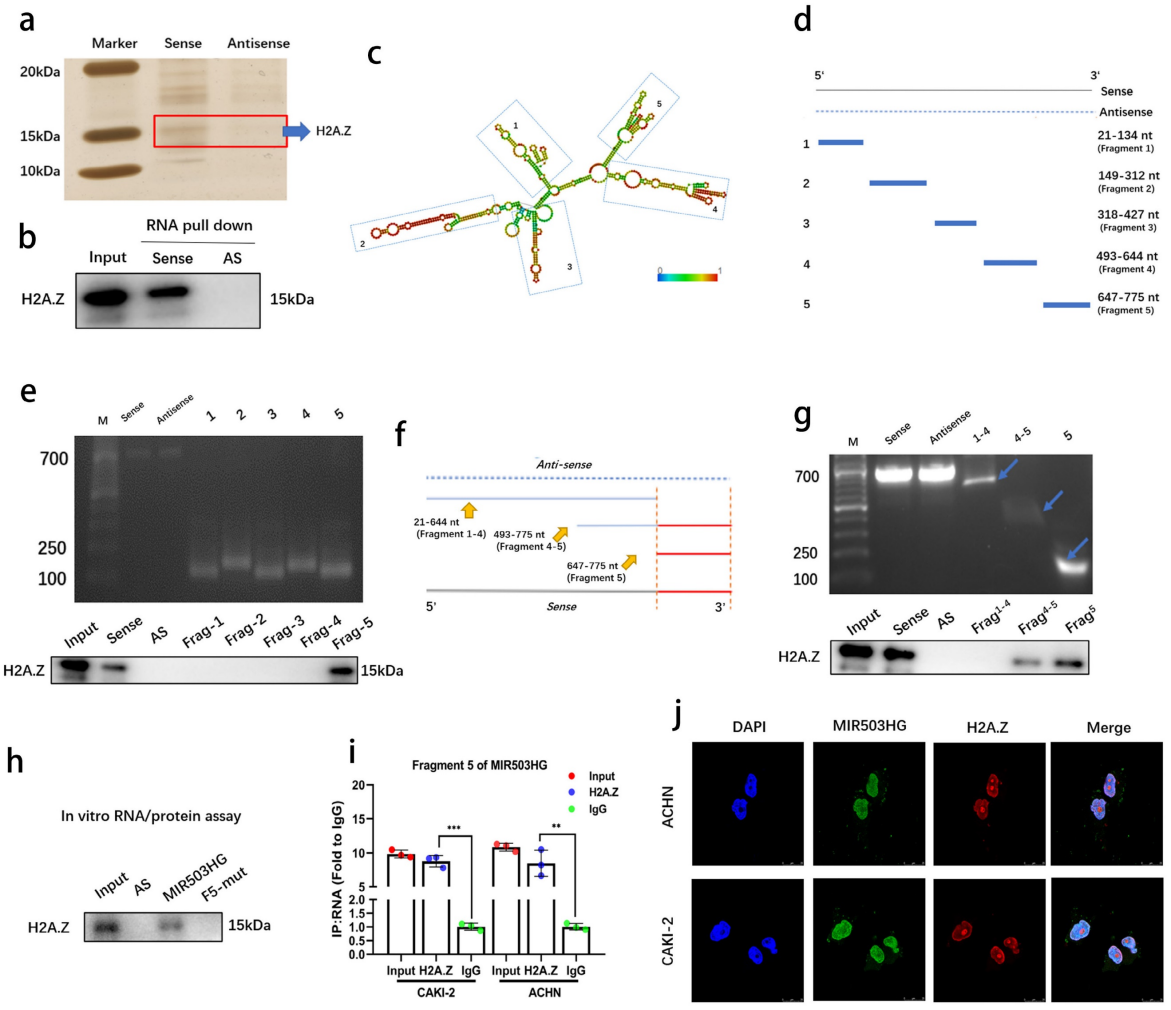
** MIR503HG directly bound with H2A.Z.** a. The silver staining image of RNA pull-down assay from pRCC tissue with MIR503HG sense and antisense RNAs. b. Representative western blots of H2A.Z pulled down by MIR503HG. c. Deletion mapping of the H2A.Z-binding domain in MIR503HG: graphic illustration of predicted secondary structure (LNCipedia, http://www.lncipedia.org). d-g. The truncations according to the stem-loop structure were shown in gel electrophoresis: In vitro transcribed full-length and deletion fragments of MIR503HG. Serial deletions of MIR503HG were used in RNA pull-down assays to identify core regions that were required for physical interaction with H2A.Z. h. RNA pull down assay between H2A.Z and indicated biotin-labeled lncRNAs. i. The enrichment of MIR503HG by the anti-H2A.Z antibody after RIP assays (IgG was used as negative). j. Representative images of IF: MIR503HG and H2A.Z co-localized in ACHN and CAKI-2 cells. The statistical difference was assessed through 2-tailed Student's t- test in i. Error bars show the standard deviation (SD) from three independent experiments. *P < 0.05; **P < 0.01; ***P < 0.001.

We conducted RNA pull-down assays to determine MIR503HG interacting proteins in pRCC. Silver staining revealed a specific protein region between 10-20 kDa in the MIR503HG pull-down samples as compared with antisense group (**Fig. 4a and Supplemental Fig. 4a**). Mass spectrometry assay was conducted in order to find the potential interacting proteins (Top 5 differential proteins identified were listed in **Table 4**). Notably, we found the potential binding partner protein, histone variant H2A.Z (**Supplemental Fig. 4b**), which has a well-known capability of regulating structure of chromatin, could participate in various biological processes by substituting the conventional histone proteins at specific locations of chromatin 27, 28. Notably, lncRNAs have also been reported to interact with H2A.Z in the regulation process of cancer cells 29.

To confirm the interaction and map the RNA-binding region of H2A.Z, western blotting was performed (**Fig. 4b**) and serial deletion assays were generated and confirmed that 647-775 nt sequences (5′-terminal of MIR503HG) was the essential part for the interaction with H2A.Z (**Fig. 4c-e**), and removal of fragment 5 (647-775 nt) abolished the MIR503HG -H2A.Z interaction (**Fig. 4f-g**). We further predicted the putative binding sites of RNA-binding protein by MEME website (**Supplemental Fig. 4c**). The results from RNA pull-down assay and RIP assay simultaneously proved the exact interactions between 647-775 nt and H2A.Z (**Fig. 4h-i**). FISH and IF assays also indicated MIR503HG and H2A.Z co-localized in the pRCC cells' nucleus (**Fig. 4j**), suggesting MIR503HG could bind with H2A.Z.

### Downregulated MIR503HG epigenetically upregulated NOTCH1 expression and promoted VEGFC secretion in pRCC

**Figure 5:**
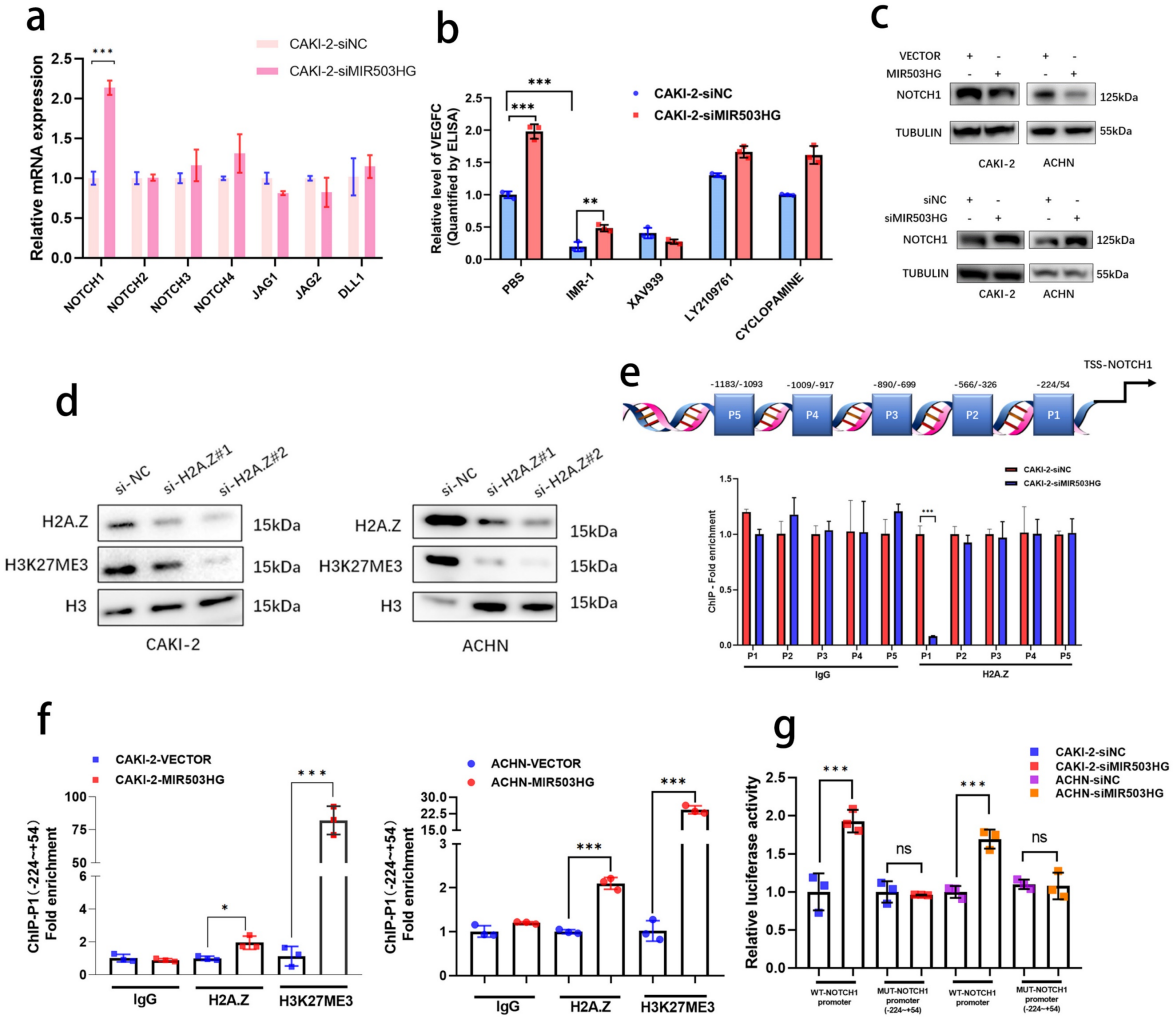
** Downregulated MIR503HG epigenetically upregulated NOTCH1 expression and promoted VEGFC secretion in pRCC.** a. qRT-PCR analysis was performed to detected the core genes involved in the NOTCH/Jagged pathway in MIR503HG-silenced CAKI-2 cells. b. ELISA for IMR-1, XAV939, LY2109761 and CYCLOPAMINE treatment on MIR503HG knockdown-induced VEGFC secretion in pRCC cells. c. Western blotting analysis evaluated the activation of the NOTCH1 by and MIR503HG overexpression (top) and MIR503HG knockdown (bottom) in pRCC cells. d. Representative image of Western blotting analysis of H3K27me3 level in H2A.Z depletion-pRCC cells e. Top: Schematic diagram of the potential binding sites in the NOTCH1 promoter; Bottom: The lysates were prepared for chromatin immunoprecipitation with H2A.Z and control antibodies, followed by examination of NOTCH1 promoter enrichment in CAKI-2 cells. f. ChIP analysis evaluated the enrichment of H2A.Z and H3K27me3 in MIR503HG-overexpression groups in pRCC cells. g. Luciferase activity was detected in MIR503HG knockdown CAKI-2 and ACHN cells transfected with NOTCH1-P1-MUT and NOTCH1-WT promoter plasmids. The statistical difference was assessed through 2-tailed Student's t- test in a, e, f and g. One-way ANOVA with Tukey's post hoc test was performed in b. Error bars show the standard deviation (SD) from three independent experiments. *P < 0.05; **P < 0.01; ***P < 0.001.

Emerging evidences revealed that non-coding RNAs were widely participated in the regulation of cellular signals in tumor progression 33, 34. In view of such phenomenon, the alteration of key genes in some important pathways related with RCC progression were detected after silencing MIR503HG expression in pRCC cells (**Fig. 5a and Supplemental Fig. 5a-d**). Notably, four pathways (Hedgehog, NOTCH/Jagged, WNT/β-catenin and TGF-β) with altered genes expression were further analyzed by ELISA assays. Rather than other pathway inhibitors, IMR-1, which specifically inhibited transcriptional activation complex on NOTCH chromatin 40, could reverse the stimulation by MIR503HG knockdown to VEGFC secretion (**Fig. 5b and Supplemental Fig. 5e**). Furthermore, consistent with the results of qRT-PCR, protein level of NOTCH1 expression were markedly increased by silencing MIR503HG expression. Conversely, levels of NOTCH1 were decreased in MIR503HG-overexpression pRCC cells (**Fig. 5c**). It is well-known that activation of NOTCH1 would promote RCC progression and metastasis 35, 36. Kaplan-Meier curves also revealed that the high level of NOTCH1 expression was associated with poor overall survival in pRCC (**Supplemental Fig. 5f**). Additionally, NOTCH1-related signaling pathway could interact and regulate the expression level of VEGF family 37-39. According to all above, we came to a conclusion that NOTCH1 was indispensable for MIR503HG-mediated VEGFC secretion in pRCC-associated lymphatic metastasis.

Given that H2A.Z was recognized as a crucial role in epigenetic modification of histone 30, 31, indeed, it epigenetically modulated target genes expression by catalyzing H3K27 trimethylation (H3K27me3) 32. We transfected pRCC cells with corresponding small interfering RNA to prove that H2A.Z knockdown in pRCC cells rescued the inhibitory effect of MIR503HG overexpression on cell viability (**Supplemental Fig. 5g**) and silencing H2A.Z observably decreased H3K27 trimethylation level (**Fig. 5d**). Out of curiosity, we began to consider whether MIR503HG could regulate expression of NOTCH1 via H2A.Z-mediated H3K27 trimethylation. ChIP assays were designed and performed with anti-H2A.Z antibody and anti-H3K27me3 antibody, showing that H2A.Z was markedly enriched between -224 and +54 nucleotides (P1) in the promoter of NOTCH1 (**Fig. 5e**), MIR503HG-overexpression may dramatically increase H3K27me3 via coordinating depositions of H2A.Z in NOTCH1 promoter region (**Fig. 5f and Supplemental Fig. 5h**). Furthermore, luciferase report assays demonstrated that MIR503HG knockdown induced the luciferase activity of WT promoter of NOTCH1, while MIR503HG overexpression markedly decreased the luciferase activity (**Fig. 5g and Supplemental Fig. 5i**).

### MIR503HG suppressed lymphatic metastasis through altering VEGFC in pRCC

To further elucidate whether MIR503HG could suppress the lymphatic metastasis by downregulating VEGFC, we developed orthotopic xenograft model by injecting cells into the subrenal capsule to determine whether antagonism of VEGFC could suppress shMIR503HG-stimulated lymphatic metastasis.

**Figure 6:**
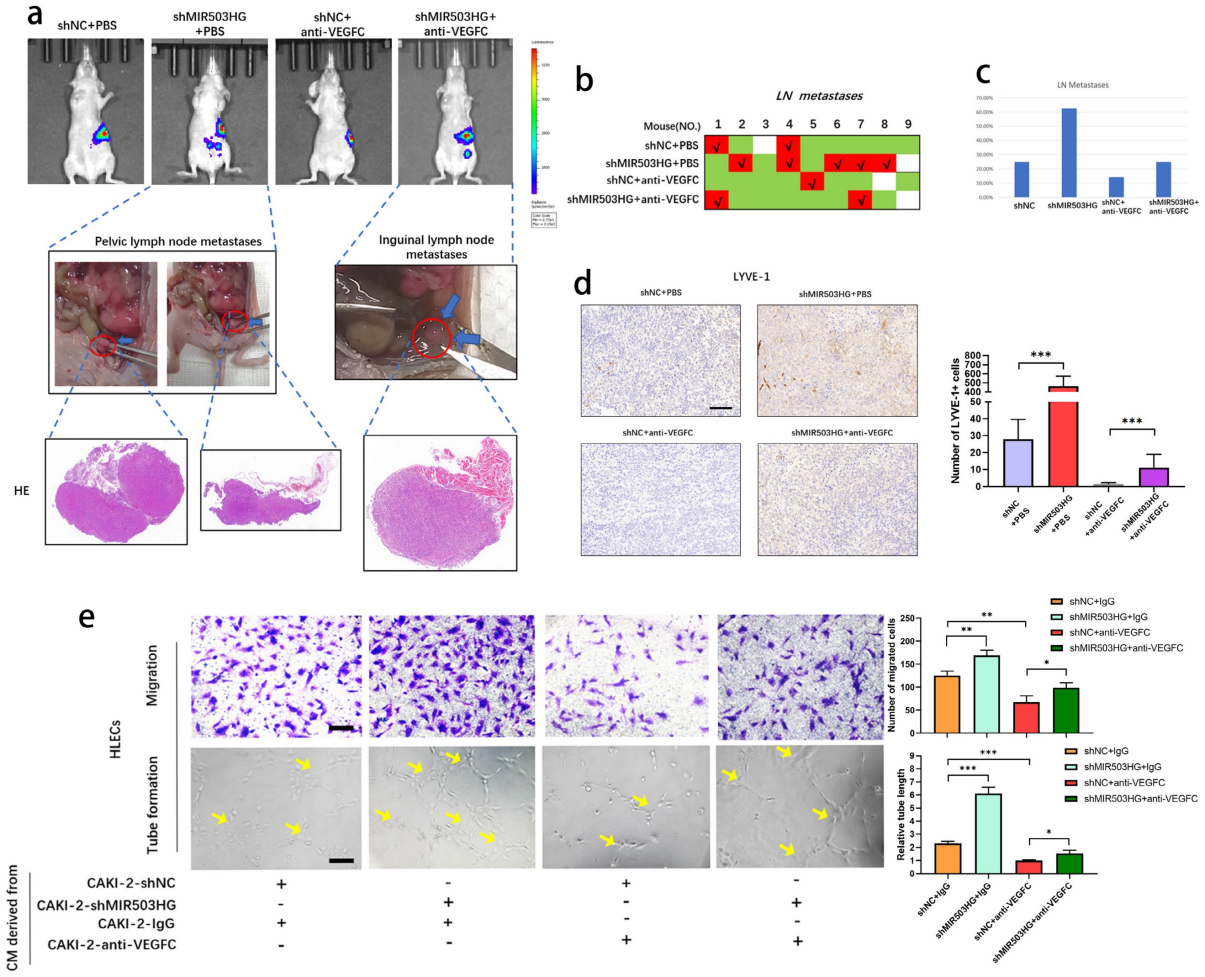
** MIR503HG suppressed lymphatic metastasis through altering VEGFC in pRCC.** a. Nude mice were orthotopically xenografted with PBS or VEGFC neutralizing antibody. LNs and primary tumor tissues were detected by bioluminescent imaging, tumor samples were enucleated and analyzed by HE staining. b-c. Metastasis diagrams of mice in the indicated groups were shown. d. Representative images and histogram analysis of LYVE‐1‐indicated lymphatic vessel density in primary tumor tissues of differently treated mice from the IHC analysis. Scale bars: 100 μm. e. Representative images and quantification of tube formation and transwell-migration for differently treated groups. Scale bars: 50 μm. The statistical difference was assessed through one-way ANOVA with Tukey's post hoc test in d and e. Error bars show the standard deviation (SD) from at least three independent experiments. *P < 0.05; **P < 0.01; ***P < 0.001.

IVIS showed that anti-VEGFC could reduce the shMIR503HG-induced metastatic capability of pRCC to LNs, and all suspicious tissues were collected and taken for histopathological observation to verify the lymphatic metastases (**Fig. 6a-c**). Consistently, IHC from LYVE-1 indicated that shMIR503HG significantly increased density of micro-lymphatic vessels in left-kidney capsule while treating with VEGFC neutralizing antibody markedly weakened shMIR503HG-stimulated lymphangiogenesis (**Fig. 6d**). On the other hand, antagonism of VEGFC significantly suppressed shMIR503HG-stimulated tube formation and migration of HLECs (**Fig. 6e**). Taken together, these results demonstrated that MIR503HG suppressed lymphatic metastasis in pRCC by regulating VEGFC.

### MIR503HG-modulated HNRNPC could maturate NOTCH1 mRNA in a m6A-dependent manner

**Figure 7:**
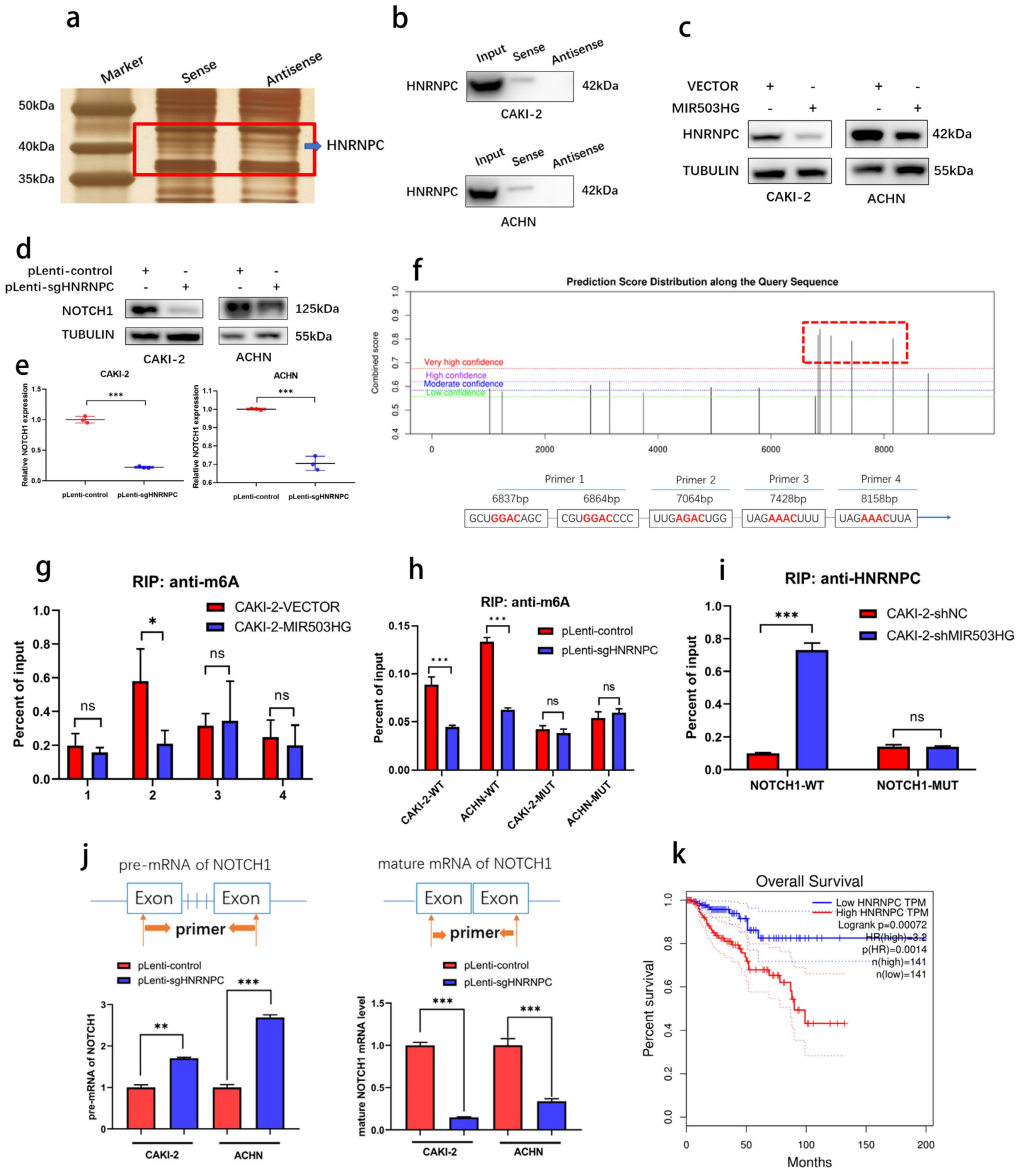
** MIR503HG-regulated HNRNPC can maturate NOTCH1 mRNA in an m6A-dependent manner.** a. Representative image of silver-stained PAGE gels revealed the differential bands between biotin-labeled MIR503HG and antisense sequence of MIR503HG. b. Western blot analysis to determine the specific association of HNRNPC with biotinylated MIR503HG. c. Western blotting analysis evaluated the expression level of the HNRNPC by MIR503HG overexpression in ACHN and CAKI-2 cells. d. Expression level of NOTCH1 protein were measured by Western blotting analysis in pLenti-control and pLenti-sgHNRNPC. e. Expression level of NOTCH1 mRNA were measured by qRT-PCR analysis in pLenti-control and pLenti-sgHNRNPC. f. The potential m6A modification sites of NOTCH1 were predicted by SRAMP. g. RIP-qRT-PCR showing MIR503HG overexpression impaired the enrichment of m6A modification in the region P2. h. The enrichment of m6A were analyzed in NOTCH1-WT and NOTCH1-P2-MUT in the control and HNRNPC KO cells. i. RIP-qRT-PCR: detecting the enrichment of HNRNPC in MIR503HG KD in the NOTCH1-P2-MUT group. j. Pre-mRNA and mature NOTCH1 mRNA were analyzed using qRT-PCR after HNRNPC KO for 48 h. k. The survival curves of high and low HNRNPC expression were plotted by the Kaplan-Meier analysis with two-tailed log-rank test. Date was from The Cancer Genome Atlas (TCGA)-KIRP dataset. The statistical difference was assessed through 2-tailed Student's t- test in e, g-j. Error bars show the standard deviation (SD) from three independent experiments. *P < 0.05; **P < 0.01; ***P < 0.001.

With silver staining, another differential binding band between MIR503HG and antisense sequence were also noticed (**Fig. 7a**). Heterogeneous nuclear ribonucleoprotein C (HNRNPC), as known as N6-methyladenosine (m6A)-associated protein, was identified (**Table 4 and Supplemental Fig. 4d**) and its interaction with MIR503HG was further validated by western blotting (**Fig. 7b**).

It surprised us that there was a negative correlation between MIR503HG and HNRNPC (**Fig. 7c**). To illustrate the possibility that MIR503HG could regulate this m6A reader to influence its downstream target NOTCH1, we successfully established HNRNPC-KO cells through CRISPR/Cas9 approach (**Supplemental Fig. 6a-c**). Positive regulatory effects of HNRNPC on NOTCH1 expression were shown by western blotting assay (**Fig. 7d-e**).

As known as the primary form of RNA modification, m6A regulates gene expression and decides eukaryote fate 41. To clarify whether NOTCH1 could be modified by m6A methylation, five m6A modification sites with very high confidence of NOTCH1 mRNA were predicted by SRAMP database (**Fig. 7f and Supplemental Fig. 6d**) and RIP-qPCR assays indicated that MIR503HG-overexpression significantly reduced HNRNPC-binding to the m6A-modified region (P2) of NOTCH1 (**Fig. 7g-h and Supplemental Fig. 6e**). Consistently, mutation of P2 abrogated the enrichment of HNRNPC in MIR503HG-silenced cells (**Fig. 7i and Supplemental Fig. 6f**).

As widely reported, HNRNPC influenced the pre-mRNA processing, transport, stability, and other aspects of mRNA metabolism 42-44. Hence, we designed primers with or without introns, and performed qRT-PCR to determine that HNRNPC-KO decreased the efficiency of NOTCH1 mRNA processing and maturation (**Fig. 7j**). Moreover, similar to NOTCH1, Kaplan-Meier analysis of overall survival of TCGA-KIRP dataset revealed that higher expression of HNRNPC was associated with poor overall survival (**Fig. 7k**).

Taken together, these findings indicated that downregulation of MIR503HG promoted maturation of NOTCH1 mRNA via HNRNPC mediating m6A-dependent manner.

### Everolimus repressed lymphatic metastasis via inducing MIR503HG

**Figure 8:**
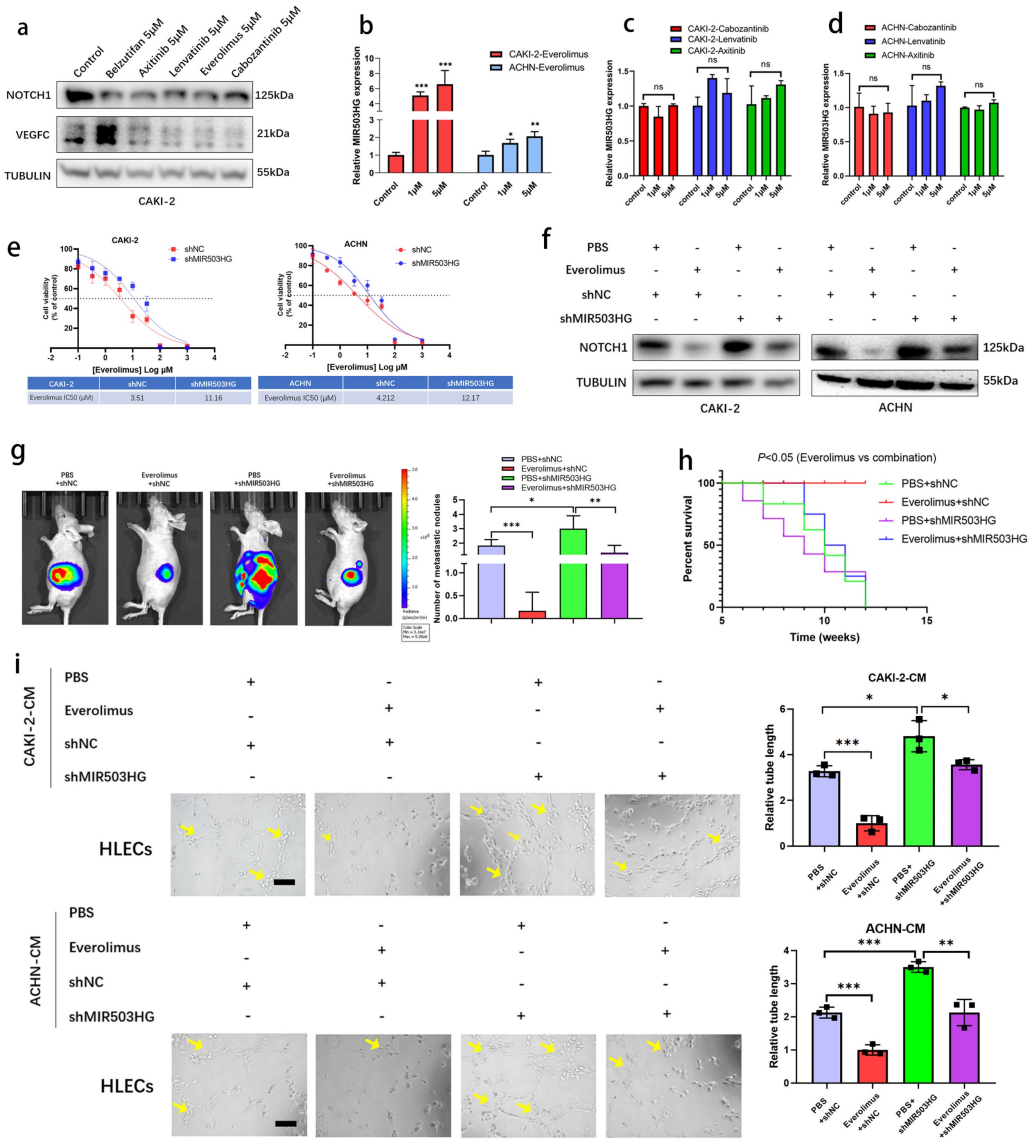
**Everolimus repressed lymphatic metastasis via inducing MIR503HG.** a. Western blotting assays were performed to determine different treatment effects (Belzutifan, Axitinib, Lenvatinib, Everolimus and Cabozantinib) on NOTCH1 and VEGFC protein expression. b-d. MIR503HG expression was measured by qRT-PCR in pRCC cells respectively treated with Everolimus (b), Cabozantinib, Lenvatinib and Axitinib (c-d). e. Dose-response curves for Everolimus in pRCC cells with or without MIR503HG knockdown. f. Western blotting assays was used to detect NOTCH1 protein expression in different treated groups. g. Orthotopic xenograft animal models were generated. Representative images of metastasis viewed by IVIS in 4 weeks after the orthotopic xenograft transplantation. h. Kaplan-Meier survival curves for 4 groups of mice were plotted. i. Representative images and quantification of tube formation for HLECs in conditioned medium from CAKI-2 or ACHN. Scale bars: 50 μm. The statistical difference was assessed through one-way ANOVA followed by Dunnett's post hoc test in b-d and followed by Tukey's post hoc test in g and i. Error bars show the standard deviation (SD) from at least three independent experiments. *P < 0.05; **P < 0.01; ***P < 0.001.

It was reported that LncRNAs enabled to predict the response to target therapy of RCC 45, 46. In present study, to identify whether MIR503HG could be the potential therapeutic target of lymphatic metastasis, several drugs, such as Everolimus, Cabozantinib, Lenvatinib and Axinitib, which were currently used for RCC treatment, were examined during research. Western blotting assay showed that VEGFC and NOTCH1 expression were downregulated by treating several drugs, respectively (**Fig. 8a and Supplemental Fig. 7a**). Notably, qRT-PCR analysis revealed that MIR503HG expression level was significantly induced in Everolimus group, while no significant difference was found in other groups (**Fig. 8b-d**). Inhibition of mTOR signaling was reported that increases the expression of p53 protein level 47. Consistently, two potential p53-binding sites in MIR503HG promoter region were detected by JASPAR database (**Supplemental Table 1**). Interestingly, qRT-PCR analysis provided additional evidences for the mechanistic connection that Everolimus-enhanced MIR503HG expression was considerably decreased in cells with concurrent p53 silence (**Supplemental Fig. 7b-c**).

It made us wonder that whether upregulating MIR503HG could enhance the sensitivity of pRCC cells to Everolimus treatment. Compared to control pRCC cells, shMIR503HG cells showed less sensitive to Everolimus (**Fig. 8e**). In addition, Everolimus treatment repressed NOTCH1 expression while depletion of MIR503HG minimized this effect (**Fig. 8f**). The pRCC cells were implanted into subrenal capsule of nude mice, consistent to survival analysis, metastasis sites assessed by IVIS confirmed that shMIR503HG reduced the therapeutic effectiveness of Everolimus in pRCC xenografts (**Fig. 8g-h**). Reducing MIR503HG expression in pRCC could partially reverse the negative effect of tumor-induced lymph-vessel formation caused by Everolimus (**Fig. 8i**). Collectively, our results suggested that MIR503HG might be a potential biomarker for pRCC treatment.

## Discussion

MIR503HG, located on chromosome Xq26.3, differentially expressed in pRCC and exhibited values of prognosis. As several studies have previously focused on its function in cancer, it generally prevented tumor cells from proliferating, encroaching and migrating 48-50. In present study, we investigated LncRNA-MIR503HG, which was inversely correlated with cell invasion, migration and lymphangiogenesis, may have a close association with lymphatic metastasis of pRCC patients.

Emerging evidences suggested that DNA methylation-mediated epigenetic alteration is one of the key processes controlling lncRNA expression and related-biological process. Moreover, this phenomenon exhibited a tissue specificity 51-54. By using bioinformatic data mining, MSP-PCR and Bisulfite sequencing, we confirmed the hypermethylation status in MIR503HG promoter. Subsequently, MIR503HG expression was proved to be negatively linked with methylation status.

In our study, we observed that MIR503HG was involved in the cellular regulation by epigenetically inducing lymphangiogenesis and invasion of pRCC by increasing H3K27me3 via H2A.Z. Yuan et al. reported that lncRNAs may function by binding with H2A.Z in cancer cells 29, the interaction was proved to regulate the acetylation of H2A.Z. Our study proposed that MIR503HG might have an effect on the formation of H2A.Z-H3K27me3 complex with targeted DNA sequences. Dynamic regulation of H2A.Z in chromatin has been implicated in transcription process 55, it was reported to facilitate the recruitment of PRC2 for appropriate installation of H3K27 trimethylation at promoters of genes controlling cell development 56. However, due to the lack of detailed structure of H2A.Z protein itself, the mechanisms through which potential DNA-binding domains to target sites across the chromatin were still unclear.

VEGFC, as a lymphatic vessel-specific growth factor, has been demonstrated that it played essential role in lymphatic metastasis, including compromising the endothelial lymphatic barrier, allowing for lymphatic invasion, and ultimately promoting lymphatic metastasis and causing treatment failure 57-59. Interdicting the VEGFC/VEGFR-3 axis has been widely reported to reduce the rate of lymphatic metastasis in a number of tumor-bearing experimental *invivo* models 60-62. Our results enhanced mechanistic understandings about pRCC-lymphatic metastasis, suggesting blockage of VEGFC reversed the effect of downregulated MIR503HG on lymphatic metastasis.

It's well-acknowledged that disorders of m6A modification in RNA are closely related to the development of solid tumor 63. Jin et al. demonstrated that HNRNPC, as known as a m6A “readers”, could mature DDX58 transcript by binding to m6A sites 64. It was also found that HNRNPC interacted with lncRNA CYTOR, and stabilized ZEB1 mRNAs by inhibiting nondegradative ubiquitination 65. However, its specific mechanism in pRCC has not been elucidated. We showed that downregulated MIR503HG could promote lymphatic metastasis by inducing HNRNPC. Moreover, HNRNPC was able to regulate downstream signal transduction through the N6-methylation of NOTCH1, which further promotes the secretion of VEGFC in pRCC cells. Our findings would provide a new perspective on the regulation of m6A modification in lymphatic metastasis.

There is no consensus among clinicians regarding which drug was the optimal treatment option for pRCC-associated lymphatic metastasis. Indeed, previous research has reported that the activation of mTOR was a widespread event in clinical samples of invading locoregional LN 66. In prostate cancer, increasing p-mTOR expression was demonstrated that has positive correlations with lymphangiogenesis and lymphatic metastasis 67. Specifically, AKT/mTOR signaling pathway acted as a critical role of promoting lymphatic metastasis in cervical cancer and medullary thyroid carcinoma 68, 69. However, further works remain to be done to define precisely how mTOR inhibitors acts in lymphatic metastasis of pRCC.

TKIs and mTORs are most common therapeutic options among the several classes of available treatments. Everolimus, as a classic mTOR inhibitor, is recommended as a second-line treatment for metastatic RCC patients previously received at least one TKI. On the pRCC front, however, there is little research evidence of everolimus treatment.

As a single-arm phase II trial with everolimus in previously untreated pRCC, the RAPTOR trial has generally received limited attention, this trial enrolled totally 88 patients in its intention-to-treat (ITT) cohort 70. Despite a very low response rate (1%), an OS of 21.4 months (95% CI, 15.4-28.4) were noteworthy. A large percentage of patients (65%) in this trial showed stable disease (SD) as a best response. As results of RAPTOR, mTOR inhibition will be a primary tactic in pRCC.

The effectiveness of doublet therapy was then discussed in light of single-agent targeted therapy's success in non-ccRCC. Hence, more possible strategies need to carry on to countering resistance mechanisms. Hutson. et al. assessed the regimen in nccRCC based on the efficacy of lenvatinib with everolimus after one line of VEGF-directed therapy 71, which means combinations may improve the treatment effectiveness of pRCC.

It's well known that ccRCC derived great benefit from VEGFR-associated multi-targeted TKIs, such as sunitinib, pazopanib, axitinib. According to our results, however, TKIs did not have any impact on MIR503HG expression level in PRCC. In our opinion, its mechanism may by realized not by directly regulating MIR503HG, but by other indirect signals. MIR503HG interacts with signaling pathways, such as inhibiting the phosphorylated proteins of RAF, MEK, ERK1/2, PI3K and AKT 72. In view of PI3K inhibitors were determined that could reverse TKIs resistance in tumor 73, and activated AKT promoting TKIs resistance in RCC 74, we speculate that MIR503HG-KO cells will be more sensitive to those TKIs than control cells.

Based on our work, we confirmed that inducing MIR503HG may have therapeutic benefit for sensitizing Everolimus treatment on pRCC and its associated lymphatic metastasis. This finding might become a unique therapeutic approach for us to suppress pRCC.

## Materials and Methods

### Clinical data and specimens

67 RNA later-preserved pRCC specimens were collected from pRCC patients underwent radical nephrectomy at Renji Hospital (Shanghai, China) between June 2013 and July 2020. The clinicopathological characteristics were summarized in detail (**Table 5**). After surgery, clinical samples, including tumor and adjacent normal tissue were immediately snap-frozen in liquid nitrogen and stored in cryogenic refrigerator at -80 °C. Formalin-fixed and paraffin-embedded (FFPE) tumor tissues were obtained and histologically confirmed by three pathologists.

### Cell culture

Cell lines involved in this study were all purchased from American Type Culture Collection (ATCC, Manassas, USA). ACHN and CAKI-2 cells were respectively cultured in Minimum Essential Medium (MEM) and McCoy's 5A media. Both of media were supplemented with 10% fetal bovine serum (FBS) (Gibco, Shanghai, China). HLECs were cultured in endothelial cell medium with 5% FBS and 1% matched growth factor (ScienCell, CA, USA). A humidified incubator was used for keeping cell lines mentioned above in the condition at 37°C with 5% CO2.

### RNA pull-down assays

Briefly, according to the manufacturer's instructions, biotinylated MIR503HG and antisense sequences were pulldown of proteins by using Magnetic RNA-Protein Pull-Down kit (Thermo Fisher Scientific, USA). RNAs were transcribed in vitro transcription with T7 and SP6 RNA Polymerase and the purified transcribed biotinylated RNAs were conjugated with magnetic beads, the conjugation was next incubated with prepared lysates at 4 °C for 24h. Beads were washed briefly to get proteins, protein products were detected by Mass spectrometry and western blotting subsequently.

### RNA immunoprecipitation (RIP) assays

RIP assays were performed to identify the RNA-binding protein by using a MagnaRIP RNA-Binding Protein Immunoprecipitation Kit (Millipore, USA) according to the manufacturer's protocols. The corresponding pRCC cells lysates were immunoprecipitated with magnetic beads conjugated with anti-H2A.Z antibody or negative control (anti-IgG) at 4 °C overnight. Phenol-chloroform was used to extract the precipitated RNAs. Finally, the eluted RNA was analyzed by qRT-PCR.

### Chromatin immunoprecipitation (ChIP) assays

ChIP assay was performed with EZ-Magna ChIP A/G (17-10086, Millipore, MA, USA) according to the manufacturer's protocol. The enrichment of the H3K27me3, H2A.Z on the promoter region of NOTCH1 was determined by using anti-H3K27me3 and anti-H2A.Z (Abcam, USA). As a negative control, normal mouse IgG were also used for immunoprecipitation. ChIP-derived DNA would be quantified using real-time qPCR analysis.

### Tube formation assay

The pRCC cells were previously cultured for 24 h. The conditioned medium was then collected from different groups. HLECs were resuspended in collected conditioned medium and cultured for 12h in 96-well plates (IBIDI, German) placed with 10 μl Matrigel (BD Biosciences, USA). Using inverted fluorescence microscopy to observe tube formation, the formed lymphatic vessels were analyzed and counted by Image J software (NIH, USA).

### Immunofluorescence (IF) staining

Cells were cultured on glass cover slips, and IF analysis was then performed as follow: pRCC cells were fixed with 4% formaldehyde for 10 min, and permeabilized with prepared 0.5% Triton X-100 for 15 min at room temperature. Blocked in blocking buffer (TBS with 0.05% tween 20) containing 10% goat serum and 1% BSA for 2 h at 25°C. The slides were then incubated with primary antibody at 4°C overnight, and then subjected to the corresponding secondary antibody incubation for 1h. After incubation, three washes with PBS were performed. Nuclei were stained with 4′,6-diamidino-2-phenylindole for 10-15 min. Images were taken with confocal microscopy (Leica Microsystems, Wetzlar, Germany). All antibodies used were listed in **Table 6**.

### RNA extraction and qRT-PCR assay

Extractions of total RNA from samples were conducted through the TRIZOL Reagent (Life Technologies). Under manufacturers' instructions, RNA was reversely transcribed into cDNA with Color Reverse Transcription Mix (EZBioscience), which was further quantified by qPCR assays to determinate the relative expression level of target gene with Color SYBR Green qPCR Master Mix (EZBioscience). And 2-∆CT method was conducted to evaluate the relative expression among genes. GAPDH were determined as internal control. All the primers used were listed in **Table 7**.

### Methylation-specific PCR (MSP-PCR)

Genomic DNA was extracted and analyzed from pRCC tissue and paired adjacent normal tissues. The MSP primers were designed from MethPrimer 2.0. purified DNA was exposed to bisulfite with a Bisulfte Conversion Kit (Tiangen, China) according to the manufacturer's protocol. After modified DNA is eluted, a nested, two-stage PCR method were followed to conduct the methylation-specific PCR. Amplified PCR products were identified by agarose gel electrophoresis (1.5%) and visualized with GelRed.

### Bisulfite genomic sequencing (BGS)

Methylated CpG sites distribution was analyzed in MIR503HG via BGS. Genomic DNA was firstly extracted from frozen pRCC samples and paired adjacent normal tissues. According to the manufacturer's protocol of Epitect Fast Bisulfite Conversion Kits (Qiagen, Germany), genomic DNA was bisulfite modified. methylated cytosine residues were retained as cytosine, wheras the unmethylated cytosine residues were converted to thymine at original sites. BGS assay was then performed to detect the distribution of methylated CpG sites of MIR503HG in each specimen.

The CpG islands of MIR503HG promoter region were predicted by MethPrimer 2.0 (http://www.urogene.org/cgi-bin/methprimer/methprimer.cgi) and EMBOSS database (http://emboss.bioinformatics.nl/cgi-bin/emboss/cpgplot) and 13 CpG sites were selected from MEXPRESS (https://mexpress.be/).

### Model of in vivo metastasis

4-to-6-week BALB/c nude mice were housed and fed under specific pathogen-free conditions. Luciferase stable expressing cells (1 × 10^6^ cells per mouse, mixed with Matrigel, 1:1) were injected orthotopically into capsule of left kidney.

We used bioluminecent imaging system (NightOWL II, LB983, Berthold Technologies, Germany) to monitor the primary tumor and potential metastases after abdominal injection of D-Luciferin at different time points (4, 8 and 12 weeks). Animal experiments were in accordance with guidelines of Renji Hospital for the use of animal in cancer research.

### Patient derived xenograft model

Fresh pRCC samples obtained from two patients underwent surgery at Renji Hospital were implanted into the left flank as subcutaneous tumor in four-week-old anaesthetized immune deficient mice. All mice were observed for maximum 120 days and the tumors from PDXs were analyzed by HE&IHC and qRT-PCR. Animal experiments were in accordance with guidelines of Renji Hospital for the use of animal in cancer research.

### CRISPR-Cas9-mediated gene deletion

The lentiCRISPR vectors containing single-guide RNAs targeting HNRNPC were designed by Beyotime. Following the instruction, sgRNAs were transfected into pRCC cells (CAKI-2 and ACHN) in order to construct HNRNPC knockout (KO) pRCC cells. The efficiency of sgRNA were evaluated by qRT-PCR and western blotting.

### Further applied methods

Immunohistochemistry (IHC), in situ hybridization (ISH) analysis, subcellular fractionation assays, dual-luciferase reporter assays, enzyme-linked immunosorbent assay (ELISA), lentivirus infection and cell transfection, transwell assays and wound-healing assays were provide in **Supplemental Methods**.

### Bioinformatics and statistical analysis

The clinical association of MIR503HG in pRCC was revealed through GEPIA database (http://gepia.cancer-pku.cn/index.html). R language (version 4.0.0, https://www.R-project.org) was used for graphic plotting with following packages: ggplot2, Complex Heatmap, and maftools. Kaplan-Meier analysis was carried out and log-rank analysis evaluate the survival rate of pRCC patients. All date performed at least in triplicate were presented as mean ± standard deviation. One-way ANOVA was used for data comparison when comparing 3 or more experimental groups, with Dunnett's post hoc test for comparing all the groups against the same control group, or Tukey's post hoc test or least significant difference for multiple comparisons with the same n in all groups. Differences among two experimental groups (numeric variables) were evaluated unpaired Student's t-test. Categorical variables were compared by Chi-square (χ2) test or Fisher's exact test. All tests were two-tailed and significant values were set at *P < 0.05, **P < 0.01, ***P < 0.001.

## Conclusion

Summarily, we propose a model that downregulated lncRNA MIR503HG promotes lymphatic metastasis through two pathways: influencing the histone modifications to alter the gene expression of NOTCH1 and upregulating HNRNPC to maturate NOTCH1 mRNA. Additionally, MIR503HG may enable us to suppress pRCC-associated lymphatic metastasis, targeting this newly identified signal pathway is worthwhile in clinic.

## Supplementary Material

Supplementary methods, figures and table.

## Figures and Tables

**Table 1:** Downregulated lncRNAs according to the GEPIA dataset.

Gene Symbol	Gene ID	Median-T	Median-N	log2FC	adjP
RP11-35J10.7	ENSG00000276668.1	0	3.015	-2.005	7.79E-40
GATA3-AS1	ENSG00000197308.8	0	3.07	-2.025	5.94E-55
CTD-2247C11.5	ENSG00000250529.1	0	3.102	-2.036	2.37E-55
H19	ENSG00000130600.15	24.975	106.944	-2.055	1.68E-15
RP11-190A12.7	ENSG00000256029.5	0	3.213	-2.075	4.91E-70
AC074286.1	ENSG00000213963.6	1.5	9.66	-2.092	2.45E-34
TCL6	ENSG00000187621.14	0.09	3.709	-2.111	1.49E-40
AC234582.1	ENSG00000279487.1	1.59	10.399	-2.138	4.51E-11
LINC00473	ENSG00000223414.2	0	3.419	-2.144	4.64E-54
RP11-397G17.1	ENSG00000226733.1	0	3.484	-2.165	3.57E-41
RP1-261G23.7	ENSG00000272114.1	8.69	42.833	-2.177	1.41E-10
LINC00671	ENSG00000213373.7	1.24	9.39	-2.214	3.18E-13
TBX2-AS1	ENSG00000267280.5	1.28	9.855	-2.251	2.29E-26
NR2F1-AS1	ENSG00000237187.8	0.845	8.185	-2.316	5.03E-52
U47924.27	ENSG00000257084.1	0	4.08	-2.345	8.77E-41
APCDD1L-AS1	ENSG00000231290.5	0.06	4.424	-2.355	1.11E-60
LINC00645	ENSG00000258548.5	0.06	4.593	-2.4	1.93E-58
RP13-895J2.6	ENSG00000277011.1	0.05	4.577	-2.409	3.83E-58
MIR503HG	ENSG00000223749.7	0.895	9.495	-2.469	1.18E-34
RP11-61L19.2	ENSG00000273335.1	0.21	5.815	-2.494	1.38E-60
RP11-573D15.8	ENSG00000197099.8	0.04	4.904	-2.505	9.35E-91
TTTY14	ENSG00000176728.7	0.155	5.606	-2.516	6.56E-21
AC006126.4	ENSG00000267045.1	0.56	8.035	-2.534	1.15E-54
HOXB-AS3	ENSG00000233101.10	2.98	22.169	-2.541	8.72E-31
RP11-849I19.1	ENSG00000263146.2	0	5.02	-2.59	1.96E-52
RP3-333A15.2	ENSG00000269933.1	0	5.028	-2.592	2.11E-75
RP11-12A20.10	ENSG00000278735.1	0	5.055	-2.598	1.01E-38
LINC00839	ENSG00000185904.11	0.18	6.355	-2.64	1.36E-26
LA16c-329F2.1	ENSG00000261399.1	0.855	10.658	-2.652	1.61E-28
RP11-752D24.2	ENSG00000248115.1	0.01	5.355	-2.654	2.72E-76
MEG3	ENSG00000214548.14	0.685	9.864	-2.689	1.1E-45
LINC01606	ENSG00000253301.5	0	5.88	-2.782	1.17E-71
AC132217.4	ENSG00000240801.1	1.615	17.157	-2.796	2.59E-18
PP7080	ENSG00000188242.4	18.91	137.324	-2.796	1.02E-32
RP11-728F11.4	ENSG00000254528.7	2.895	26.88	-2.839	3.4E-44
RP4-655J12.4	ENSG00000233154.5	0.13	7.15	-2.85	1.46E-83
RP11-35N6.1	ENSG00000148123.14	0.435	9.479	-2.868	1.54E-35
RP13-452N2.1	ENSG00000242048.3	0.02	6.708	-2.918	3.78E-82
RP11-334E6.12	ENSG00000263873.1	3.75	37.866	-3.033	1.29E-21
LINC01055	ENSG00000235366.2	0	8.119	-3.189	1.78E-67
AC093326.1	ENSG00000223985.1	0.1	9.219	-3.216	7.79E-46
LINC01187	ENSG00000249601.2	0	8.707	-3.279	1.14E-93
RP11-690G19.4	ENSG00000265460.6	0	9.751	-3.426	4.1E-102
RP4-568C11.4	ENSG00000274173.1	0.04	14.147	-3.864	4.33E-66
RP11-445F12.1	ENSG00000277268.1	0.06	14.535	-3.873	6.05E-46
C16orf89	ENSG00000153446.15	0.39	20.448	-3.948	2.01E-50
CH507-152C13.3	ENSG00000276076.4	0.075	36.816	-5.137	3.69E-71
LINC00982	ENSG00000177133.10	0.07	56.767	-5.755	5.2E-121

**Table 2:** Clinicopathologic characteristics of two PDX models.

Patient ID	Age	Sex	T stage	N stage	Tumor size	Metastasis site	Relative MIR503HG expression
KC6	59	Female	T1b	0	>4cm	None	High
KC67	37	Female	T3a	0	≤4cm	Paraaortic LN/ Retroperitoneal LN	Low

**Table 3:** The detailed information of 13 CpG sites in MIR503HG promoter based on TCGA-KIRP data.

Composite Element	Chromosome	Start	End
cg00719224	X	134547488	134547537
cg07924363	X	134546783	134546832
cg08141518	X	134546895	134546944
cg18719157	X	134546846	134546895
cg01807688	X	134546661	134546710
cg18412777	X	134546636	134546685
cg23245720	X	134546519	134546568
cg07776419	X	134546447	134546496
cg22955387	X	134546438	134546487
cg07194250	X	134546433	134546482
cg01972979	X	134546416	134546465
cg20978230	X	134546340	134546389
cg04109661	X	134546170	134546219

**Table 4:** Results from biotinylated MIR503HG RNA pull-down experiments. Top 5 differential proteins were identified by Mass spectrometry, respectively.

NO.	Reference	Coverage	Unique Peptides	Peptides
1	**H2A.Z**	13.64	2	17
2	TPIS	21.29	4	4
3	EXOS7	8.25	1	1
4	GAPD	5.38	1	1
5	PRDX1	19.59	2	2
1	TUBA1C	49.86	1	21
2	RBMXL1	42.14	6	25
3	**HNRNPC**	41.73	11	12
4	PSMD7	37.02	3	3
5	ALDOC	39.45	3	1

**Table 5:** Correlation between MIR503HG expression and clinicopathologic characteristics in pRCC patients.

Product	Source	No. of Catalogue
**Primary antibody:**		
***Western blot:***		
anti-β-TUBULIN	Cell Signaling Technology	#2146
anti-NOTCH1	Abcam	ab52627
anti-VEGFC	Cell Signaling Technology	#2445
anti-H2A.Z	Abcam	ab150402
anti-YTHDC1	Abcam	ab259990
anti-HNRNPC	Proteintech	11760-1-AP
Anti-LAMIN B1	Abcam	ab16048
***IF:***		
anti-H2A.Z	Abcam	ab150402
***IP:***		
anti-H2A.Z	Abcam	ab150402
anti-m6A	Abcam	ab284130
anti-H3K27ME3	Abcam	ab192985
Anti-HNRNPC	Proteintech	11760-1-AP
***IHC:***		
anti-LYVE-1	Abcam	ab218535
**Secondary antibody:**		
***Western blot:***		
anti-rabbit IgG-HRP	Cell Signaling Technology	7074
anti-mouse IgG-HRP	Cell Signaling Technology	7076
***IHC:***		
anti-rabbit IgG-HRP	Proteintech	SA00001-2
anti-mouse IgG-HRP	Proteintech	SA00001-1
***IF:***		
anti-rabbit IgG-HRP	Panovue Biological	0015001010

Abbreviations: T stage = tumor stage. ^†^ Chi-square test, ^*^ *p* <0.05, ^**^ *p* <0.01

**Table 6:** Antibodies used in the experiments.

Product	Source	No. of Catalogue
**Primary antibody:**		
***Western blot:***		
anti-β-TUBULIN	Cell Signaling Technology	#2146
anti-NOTCH1	Abcam	ab52627
anti-VEGFC	Cell Signaling Technology	#2445
anti-H2A.Z	Abcam	ab150402
anti-YTHDC1	Abcam	ab259990
anti-HNRNPC	Proteintech	11760-1-AP
Anti-LAMIN B1	Abcam	ab16048
***IF:***		
anti-H2A.Z	Abcam	ab150402
***IP:***		
anti-H2A.Z	Abcam	ab150402
anti-m6A	Abcam	ab284130
anti-H3K27ME3	Abcam	ab192985
Anti-HNRNPC	Proteintech	11760-1-AP
***IHC:***		
anti-LYVE-1	Abcam	ab218535
**Secondary antibody:**		
***Western blot:***		
anti-rabbit IgG-HRP	Cell Signaling Technology	7074
anti-mouse IgG-HRP	Cell Signaling Technology	7076
***IHC:***		
anti-rabbit IgG-HRP	Proteintech	SA00001-2
anti-mouse IgG-HRP	Proteintech	SA00001-1
***IF:***		
anti-rabbit IgG-HRP	Panovue Biological	0015001010

**Table 7:** Primers and probes used in the experiments.

Gene	Sequence (5'-3')	Application
MIR503HG	F: CCCCCAACAAAGGAACACTA R: ACTTGGGTGGTTTTCAATGC	qRT-PCR
AC006126.4	F: TTCAGGGCTGGAGGAAGAAG R: GGAGGCCAAAATCTGCTGAG	qRT-PCR
RP11-334E6.12.1	F: CAGCGAAGCGGGGGCAGGG R: GGCCTTGGGGCATGACCCAG	qRT-PCR
NOTCH1	F: ATGCAGAACAACAGGGAGGA R: ACCAGGTTGTACTCGTCCAG	qRT-PCR
NOTCH2	F: TTGTGACATAGCAGCCTCCA R: CATTCTGGCAGGGCTGATTC	qRT-PCR
NOTCH3	F: TTGTGACATAGCAGCCTCCA R: CATTCTGGCAGGGCTGATTC	qRT-PCR
NOTCH4	F: TGTGAGAAAGGCTGCAACAC R: GGGCTGCTCTCTCCTGATAG	qRT-PCR
JAGGED1	F: CCGAGGTCCTATACGTTGCT R: CCAGCCTTCCATGCAAGTTT	qRT-PCR
JAGGED2	F: CAACCCCTGTGTGAATGGTG R: ATTGTAGCAAGGCAGAGGGT	qRT-PCR
DLL1	F: CAGAAAGACTCATCAGCCGC R: CAGCCCTCTCCGTAGTAGTG	qRT-PCR
U1	F: TGAAGGCGCTTTTCTCATGG R: CAGGGGAAAGCACGAACG	qRT-PCR
WNT9A	F: CCTCTATGCCATCTCCTCGG R: TTCCTTGACGAACTTGCTGC	qRT-PCR
WNT5A	F: TCCTCCGTGTTGTGATGTGA R: GATACGCTGCAACACCTCTG	qRT-PCR
WNT3A	F: GGACAAAGCTACCAGGGAGT R: ACCATCCCACCAAACTCGAT R: AGAAGGTGACAAGAGGCTCC	qRT-PCR
FZD5	F: TAGCGGTTTTGTGTTCAGCC R: TGCCATCTCACCAGCCTAAA R: AGAAGGTGACAAGAGGCTCC	qRT-PCR
RNF43	F: AGTGGGGAGACTAGCACCTA R: ATGAATCGGAGCCTAGCCTC R: AGAAGGTGACAAGAGGCTCC	qRT-PCR
ZNRF3	F: TCTGAAGACCCGCTCAAGAG R: GAGACCACGACGAAGAAAGC R: AGAAGGTGACAAGAGGCTCC	qRT-PCR
ACTB	F: TGGCACCACACCTTCTACAA R: CCAGAGGCGTACAGGGATAG	qRT-PCR
GAPDH	F: CATGAGAAGTATGACAACAGCC R: AGTCCTTCCACGATACCAAAGT R: AGTCCTTCCACGATACCAAAGT	qRT-PCR
SUFU	F: GGCTGATAACTGACATGCGG R: GTGGAAGGACAGGTTTGCTG	qRT-PCR
SMO	F: TCCTCACTGTGGCAATCCTT R: GGCAGCTGAAGGTAATGAGC	qRT-PCR
SHH	F: ATGAAGAAAACACCGGAGCG R: GCAGTGGATATGTGCCTTGG	qRT-PCR
DHH	F: TTCCGGGAAATGCAACTGTG R: TCCACAGCCACAAATGAAGC	qRT-PCR
IHH	F: CTCGCCTACAAGCAGTTCAG R: CCTGTGTTCTCCTCGTCCTT	qRT-PCR
TGF-β1	F: CTTTCCTGCTTCTCATGGCC R: TCCAGGCTCCAAATGTAGGG	qRT-PCR
TGF-β2	F: CCTTCTTCCCCTCCGAAACT R: ATGGCATCAAGGTACCCACA	qRT-PCR
TGF-β3	F: AGATCCTTCGGCCAGATGAG R: GTCATCCTCATTGTCCACGC	qRT-PCR
BMP2	F: AATGCAAGCAGGTGGGAAAG R: GCTGTGTTCATCTTGGTGCA	qRT-PCR
BMP6	F: AGAAGAAGGCTGGCTGGAAT R: GACTCCATCCCTTGTCACCA	qRT-PCR
SMAD2	F: CTTTGTGCAGAGCCCCAATT R: CTTGTTACCGTCTGCCTTCG	qRT-PCR
SMAD3	F: GCAGAACGTCAACACCAAGT R: CGAACTCACACAGCTCCATG	qRT-PCR
MST1	F: CCAGGGACCAAGTGTGAGAT R: GTTGTGGGTAAAGCAGGCAA F	qRT-PCR
MST2	F: GGCAAGCTGACACATCGATT R: GCGTTCGCAACTGTGTAGAT	qRT-PCR
YAP1	F: CACAGCTCAGCATCTTCGAC R: TATTCTGCTGCACTGGTGGA F	qRT-PCR
YAP2	F: TCCACCAGTGCAGCAGAATA R: CACTGGAGCACTCTGACTGA	qRT-PCR
TEAD3	F: FGCAAGATGTACGGCCGAAAT R: GGTGGGCTGAACTTGTTCTG	qRT-PCR
TEAD4	F: GTCTCAGGGTTTTGGCAAGG R: CAGGAAGGCAGAGAACTCCA	qRT-PCR
NOTCH1-P1	F: AACGAGAAGTAGTCCCAGGC R: TCCCTCAGAGCATAGCAGC	ChIP
NOTCH1-P2	F: GGATGTCTGGCGGCGATC R: GGCGACTGAAATTTGCATGTG	ChIP
NOTCH1-P3	F: GACCCGTTTGTGCTTTCTGG R: CGAGCACTTGACCGCGAG	ChIP
NOTCH1-P4	F: ATCGCACTCACCACCCTG R: TAAGCGCCAGGAGTCCAAAA	ChIP
NOTCH1-P5	F: GGTGTGAGATTTGTCCTGC R: CGAAAGGCTGGGAGGGAC	ChIP
si-*MIR503HG*	sense GACAAGAACUAAAGUGGAATT antisense UUCCACUUUAGUUCUUGUCTT	si-RNA
si-*MIR503HG#2*	sense CACUCAGUGUAAUAUUAUATT antisense UAUAAUAUUACACUGAGUGTT	si-RNA
si-*MIR503HG#3*	sense CAGAAGACUCCUGUUUCAATT antisense UUGAAACAGGAGUCUUCUGTT	si-RNA
si-AC006126.4	sense GAAGAAUCUGGGAGUGUCATT antisense UGACACUCCCAGAUUCUUCTT	si-RNA
si-AC006126.4#2	sense CCCUGAGGUCCUCUCGGAATT antisense UUCCGAGAGGACCUCAGGGTT	si-RNA
si-AC006126.4#3	sense GAUACGCAGGAGCUCAGCATT antisense UGCUGAGCUCCUGCGUAUCTT	si-RNA
si-RP11-334E6.12.1	sense GGUCAAACCUGCAUCUUCATT antisense UGAAGAUGCAGGUUUGACCTT	si-RNA
si-RP11-334E6.12.1#2	sense GCUGGAGAUGCUCAGUUCUTT antisense AGAACUGAGCAUCUCCAGCTT	si-RNA
si-RP11-334E6.12.1#3	sense GGAGUGCUGUCAGGACAGATT antisense UCUGUCCUGACAGCACUCCTT	si-RNA
Mature mRNA of NOTCH1	F: TGTGCACTGTGAGATCAACG R: TCGCAGGGATTGGACAGG	qRT-PCR
Pre-mRNA of NOTCH1	F: TCAACGCCGTAGATGACCTG R: CCGTTCTTCAGGAGCACAAC 5'-DIG labeled and 3'-DIG labeled	qRT-PCR

## Abbreviations

GEPIA: Gene Expression Profiling Interactive Analysis
GAPDH: glyceraldehyde-3 phosphate dehydrogenase
VEGFC: vascular endothelial growth factor C
RCC: renal cell carcinoma
pRCC: papillary renal cell carcinoma
LN: lymph node
KIRP: kidney renal papillary carcinoma
KIRC: kidney renal clear cell carcinoma
KICH: kidney chromophobe
CCLE: Cancer Cell Line Encyclopedia
CPG: cytosine-guanine
FFPE: formalin fixed paraffin-embedded
PDX: patient derived xenograft
HLEC: human lymphatic endothelial cell
m6A: N(6)-methylation of adenosine
HNRNPC: heterogeneous nuclear ribonucleoprotein C
IVIS: In-Vivo Imaging System
TKI: tyrosine kinase inhibitors
