# Supplementary material for: Loss of MIR503HG facilitates papillary renal cell carcinoma associated lymphatic metastasis by triggering NOTCH1/VEGFC signaling
Source: Int J Biol Sci. 2023 Jun 19;19(10):3266–84. doi: 10.7150/ijbs.83302 (PMC10321273; doi:10.7150/ijbs.83302)
Supplement: Supplementary file 1 — Supplementary methods, figures and table. [file ijbsv19p3266s1.pdf]

Supplemental Material

Supplemental Methods

Immunohistochemistry (IHC) analysis

IHC was conducted to find the proteins in primary pRCC tissues, nude tumors in situ, or LNs. Briefly, the sections were heated at 55-60 °C for two hours, deparaffinized using xylene, and rehydrated with gradient alcohol (95%, 85% and 75%, respectively). The sections were then immerged in EDTA (Ethylene Diamine Tetraacetic Acid) buffer to retrieval antigen, which was heated in a microwave for 6 minutes on medium-high and for 15 minutes on medium-low, respectively. Samples were next exposed with 3% hydrogen peroxide for 15 min, in order to inhibit endogenous peroxidase activity, and followed by goat serum for 30 min at 25°C. Subsequently, the sections were treated with primary antibody for an overnight period at 4 °C overnight and secondary antibodies at room temperature for an interval of 30 to 60 min, respectively. After staining with 3,3'-Diaminobenzidine and counterstaining with hematoxylin, Nikon eclipse 80i microscope (Nikon, Tokyo, Japan) were used to capture images of sections.

In situ hybridization (ISH) analysis

The pRCC tissue samples were probed for MIR503HG using the techniques below. In a nutshell, as described in IHC analysis, the tissue in the sections were extensively digested with pepsin before being deparaffinized and rehydrated. The sections were incubated with anti-biotin antibody at 37 °C for two hours after being hybridized with the biotin labeled-MIR503HG oligonucleotide probe at 37 °C overnight. The sections were stained with BCIP/NBT and nuclear fast red was utilized as counterstain. Images were captured by Nikon eclipse 80i (Nikon, Tokyo, Japan).

Subcellular fractionation assays

Subcellular fractionation assays were carried out in according to the instruction of manufacturers (PARIS^{TM} Kit) to identify cell location of target genes. GAPDH and U1 were used as internal references.

FISH

ACHN and CAKI-2 cells were pretreated with 0.5% Triton X-100 in order to permeabilize the cells as describing in the IF assays before planted in the confocal dish. After that, the FITC-labeled MIR503HG probes were utilized to hybridize at 4 °C overnight, and cell nuclei were then stained with DAPI at room temperature for at least 10 min. Confocal microscopy was used to take the pictures.

Enzyme-linked immunosorbent assay (ELISA)

ELISA assays were carried out under the instructions of Human VEGF-C ELISA Kit (Abcam). Adding the required amount of culture media from MIR503HG-knockdown pRCC cells, VEGF-C antibody was incubated at room temperature for 1 h. The plates rinsed 6 times with PBST and treated with TMB Development Solution for 15 min, then 100 μL Terminate Solution was added to stop the total reaction. The OD at 450 nm and 570nm were then measured.

Dual-luciferase reporter assays

The Dual-luciferase reporter assays were used to identify the mechanism by which MIR503HG control NOTCH1 transcription. Experimental procedures were accord with the instructions of Dual-Luciferase Reporter Assay System (Promega, Madison, WI, USA). Briefly, the various pretreated groups were collected and washed with PBS for three times, then lysed for 20 min in PBL lysis buffer.

Supernatants were gathered and put onto 96-well plates (10 μl each well), 100 μl Luciferase Assay Reagent II was added for exactly two seconds. Subsequently, Thermo's MK3 plates reader (Thermo, USA) was used to record luciferase intensity and renilla luciferase intensity (control).

The siRNAs and lentivirus infection and cell transfection

siMIR503HG, siRP11-334E6.12 and siAC006126.4 were purchased from Tsingke.

shMIR503HG expressing a short hairpin RNA targeting MIR503HG and overexpressing MIR503HG was packaged into lentivirus vector (Obio, Shanghai, China), then transfected into HEK293T cells in order to amplify target virus. Purified

Lentiviruses were harvested to infect CAKI-2 and ACHN cells to construct the stable knockdown or overexpression cell lines, in which puromycin was used to select the successfully infected cells for 14 days. Transfection efficiency was evaluated by western blotting and qRT-PCR assays.

Transwell assays

Briefly, cells were resuspended in FBS-free medium. 600 μl of culture medium supplemented with 5% FBS was added to the lower chamber, and 300 μl suspension containing 2×10^5 cells were seeded into the top chamber (24-well insert, 8 μm, Corning, USA). The chambers were then incubated at 37°C in a humidified environment for 12 hours. Cells were stained with crystal violet for an additional 20 minutes after being fixed in 4% paraformaldehyde for 30 minutes. Image from an inverted microscope were taken, and invading cells at three random fields by Image J software.

Wound healing assays

A straight scratch was made when cells grown to 80% confluency in the plate with a 200 μl pipette tip and the plate was then incubated for 24 hours at 37°C. Inverted microscope was used to capture images.

Serial deletion analysis

In order to discover sequence of MIR503HG involved in its interaction with H2A.Z, serial deletion analysis was carried out. Following this, pcDNA3 vectors containing various truncated fragments were constructed and subsequently utilized to conduct RNA pull-down analysis.

Cell viability analysis

Approximately 2000 cells/well were seeded in 96-well plates with 100 μl of complete culture medium. At 24 h after incubation, the medium was removed and 100 μl fresh complete culture medium containing 10 μl CCK-8 was added. Absorbance of the wells was then detected using a spectrophotometer (Thermo Scientific, Pittsburgh, PA, USA). Absorbance of each group were measured at 450nm to determine the cell viability.

Supplemental Figures

Figure S1. Efficiency of gain-of-function or loss-of-function experiments in the LncRNA knockdown or overexpression cells.

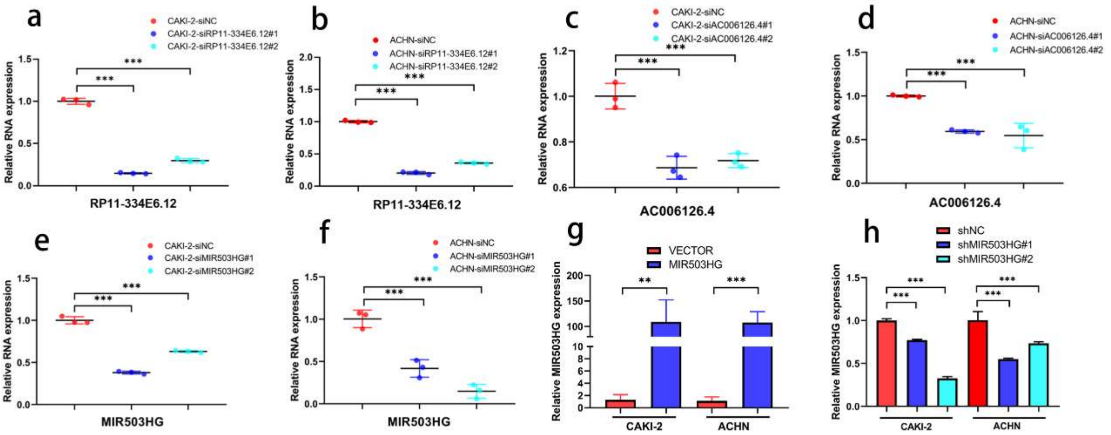

a-f. qRT-PCR analysis expression level of RP11-334E6.12 (a-b), AC006126.4 (c-d) and MIR503HG (e-f) in pRCC cells treated with relative siRNA. g-h. qRT-PCR analysis of efficiency of overexpression of MIR503HG (g) and shRNA-MIR503HG (h) in pRCC cells. The statistical difference was assessed through one-way ANOVA followed by Dunnett's post hoc test in a-f and h. The two-tailed Student's t-test was used in g. Error bars show the standard deviation (SD) from three independent experiments. $*P < 0.05$; $**P < 0.01$; $***P < 0.001$.

Figure S2. MIR503HG overexpression inhibit migration and invasion of pRCC cells *in vitro*.

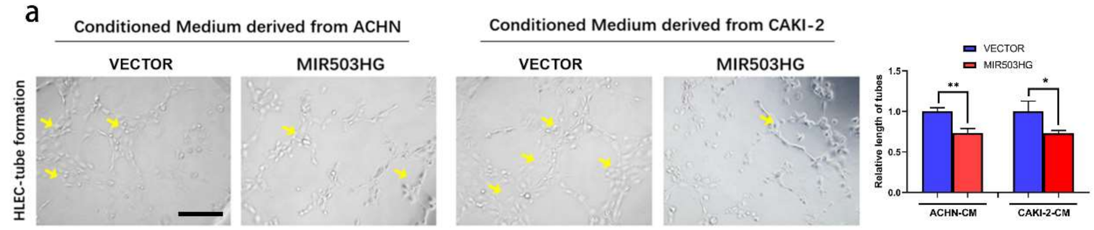

a. Representative images and quantification of tube formation for HLECs treated with culture media from vector and MIR503HG overexpression cells. The statistical difference was assessed through 2-tailed Student's t- test in a. Error bars show the standard deviation (SD) from three independent experiments. *$P < 0.05$; **$P < 0.01$; ***$P < 0.001$.

Figure S3. LncRNA MIR503HG was regulated by DNA methylation in pRCC.

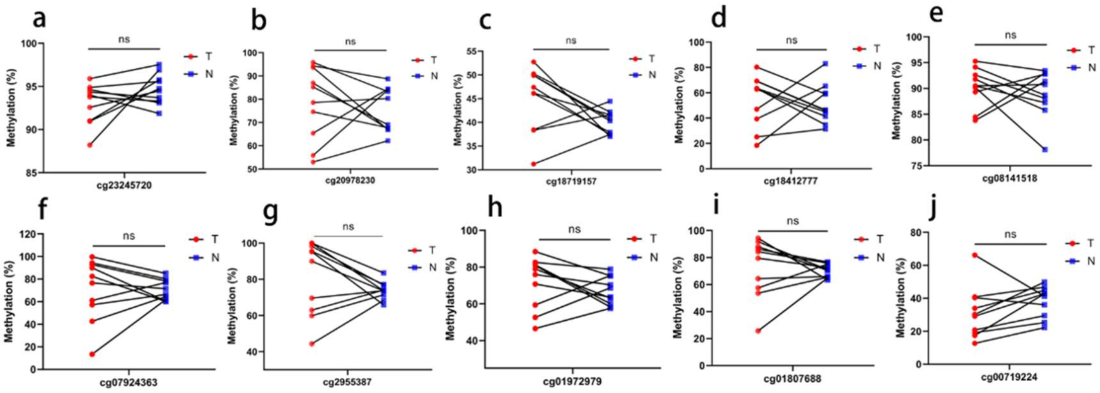

a-j. Representative sequencing analysis: the methylation level of 10 CpG sites between pRCC and normal tissue samples. The Wilcoxon Signed rank test was used in a-j.

Figure S4. MIR503HG directly interacted with RNA-binding proteins.

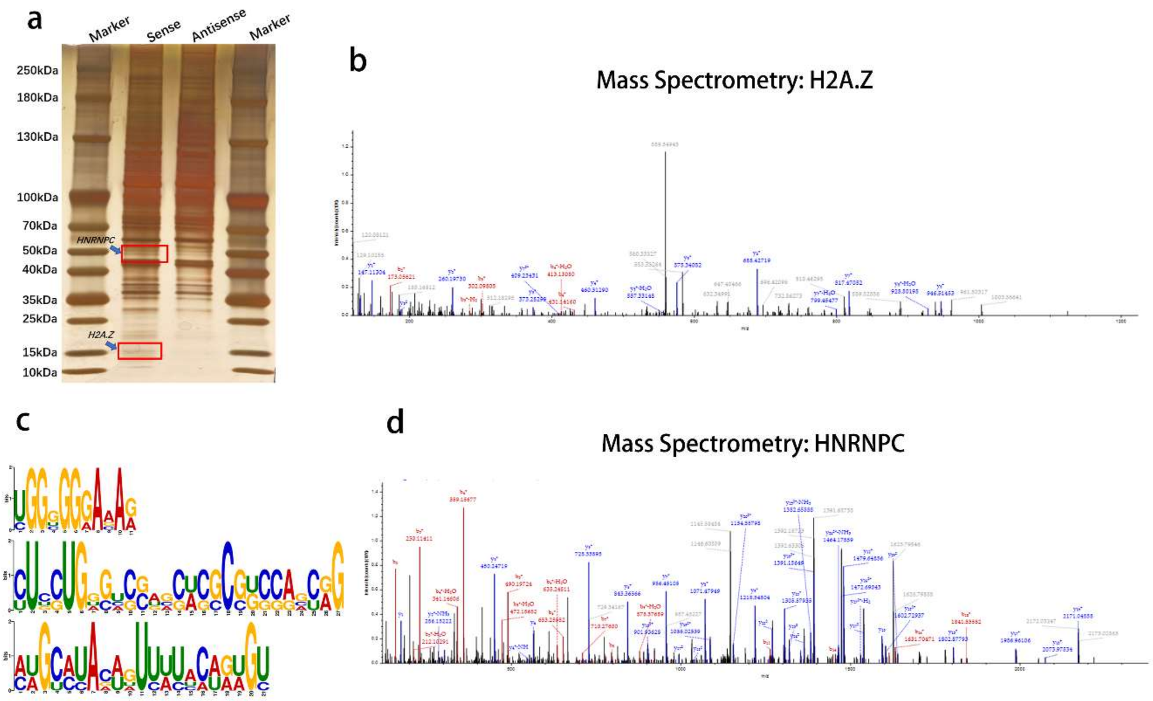

a. Full uncut original pictures of silver staining. b. Peptides of H2A.Z identified by Mass spectrometry analysis. c. Potential binding sequence motifs of MIR503HG were predicted by MEME (a sequence motif finding tool). d. Unique peptides of HNRNPC was identified by Mass spectrometry.

Figure S5. MIR503HG suppressed NOTCH1 expression via H2A.Z-associated H3K27me3 modification on NOTCH1 promoter.

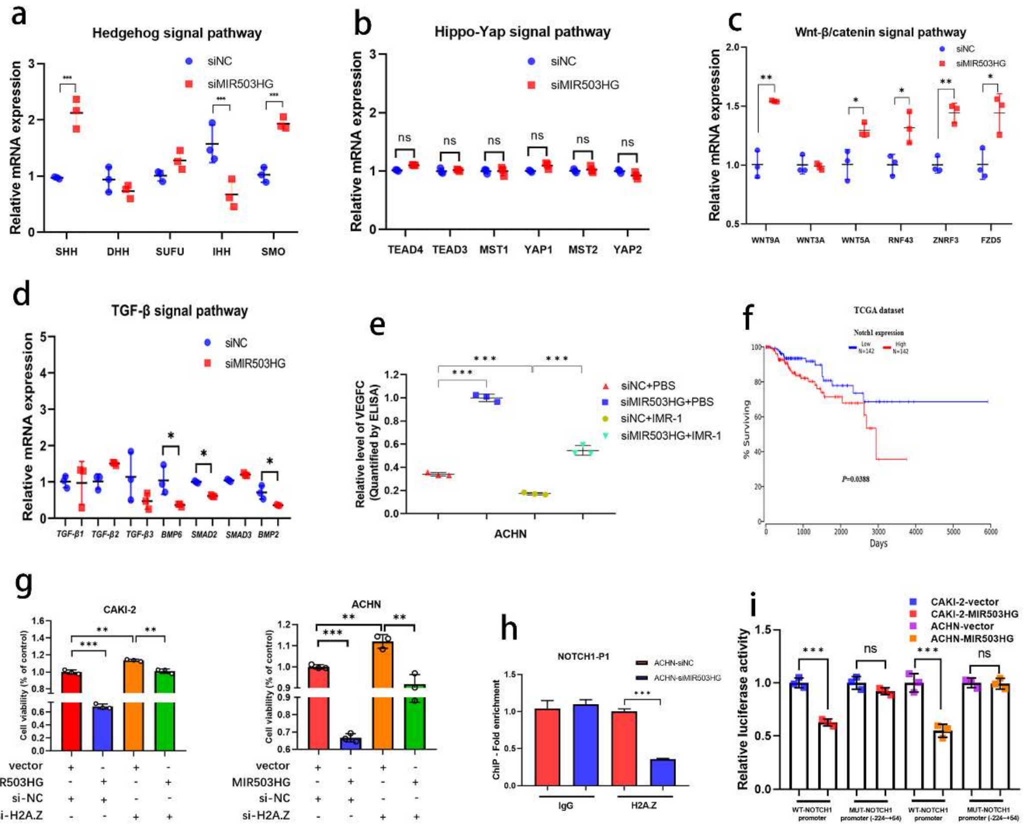

a-d. Core genes involved in the key signaling pathways were detected in MIR503HG knockdown CAKI-2 cells. Including Hedgehog (a), Hippo-Yap (b), Wnt/β-catenin (c) and TGF-β (d) pathways were analyzed using qRT-PCR analysis. e. ELISA for IMR-1-treatment on MIR503HG silenced-induced VEGFC secretion by ACHN cells. f. The survival curves of high and low NOTCH1 expression were plotted by the Kaplan-Meier analysis with two-tailed log-rank test. Date was from The Cancer Genome Atlas KIRP dataset. g. CCK-8 assay indicated that H2A.Z knockdown rescued MIR503HG overexpression induced proliferation inhibition in pRCC. h. ChIP assays were performed to detect the MIR503HG-associated chromatin region (P1) of NOTCH1. i. Dual luciferase activity in pRCC cells co-transfected with control vector and MIR503HG overexpression plasmid. The statistical difference was assessed through 2-tailed Student's t- test in a-d, h-i. One-way ANOVA followed by Tukey's post hoc test in e and g. Error bars show the standard deviation (SD) from at least three independent experiments. **P* < 0.05; ***P* < 0.01; ****P* < 0.001.

Figure S6. MIR503HG-regulated HNRNPC can maturate NOTCH1 mRNA.

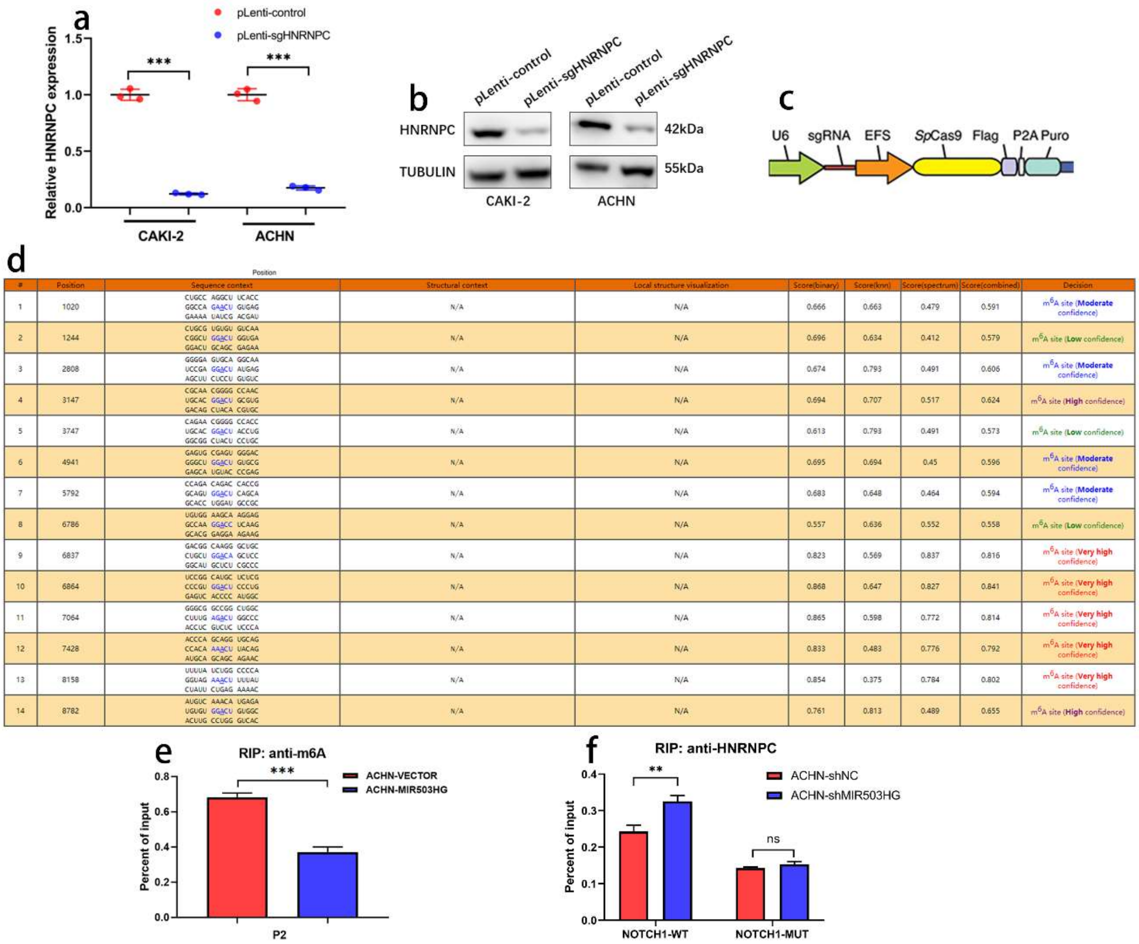

#	Position	Sequence context	Structural context	Local structure visualization	Score(binary)	Score(knn)	Score(spectrum)	Score(combined)	Decision
1	1020	CUGCC AGGCU UCACC<br>GGCCA GAACU GUGAG<br>GAAAA UAUCG ACGAU	N/A	N/A	0.666	0.663	0.479	0.591	m^6A site (Moderate confidence)
2	1244	CUGCG UGUGU GUCAA<br>CGGCU GGACU GGUGA<br>GGACU GCAGC GAGAA	N/A	N/A	0.696	0.634	0.412	0.579	m^6A site (Low confidence)
3	2808	GGGGA GUGCA GGCAA<br>UCCGA GGACU AUGAG<br>AGCUU CUCCU GUGUC	N/A	N/A	0.674	0.793	0.491	0.606	m^6A site (Moderate confidence)
4	3147	CGCAA CGGGG CCAAC<br>UGCAC GGACU GCGUG<br>GACAG CUACA CGUGC	N/A	N/A	0.694	0.707	0.517	0.624	m^6A site (High confidence)
5	3747	CAGAA CGGGG CCACC<br>UGCAC GGACU ACCUG<br>GGCGG CUACU CCUGC	N/A	N/A	0.613	0.793	0.491	0.573	m^6A site (Low confidence)
6	4941	GAGUG CGAGU GGGAC<br>GGGCU GGACU GUGCG<br>GAGCA UGUAC CCGAG	N/A	N/A	0.695	0.694	0.45	0.596	m^6A site (Moderate confidence)
7	5792	CCAGA CAGAC CACCG<br>GCAGU GGACU CAGCA<br>GCACC UGGAU GCCGC	N/A	N/A	0.683	0.648	0.464	0.594	m^6A site (Moderate confidence)
8	6786	UGUGG AAGCA AGGAG<br>GCCAA GGACC UCAAG<br>GCACG GAGGA AGAAG	N/A	N/A	0.557	0.636	0.552	0.558	m^6A site (Low confidence)
9	6837	GACGG CAAGG GCUGC<br>CUGCU GGACA GCUCC<br>GGCAU GCUCU CGCCC	N/A	N/A	0.823	0.569	0.837	0.816	m^6A site (Very high confidence)
10	6864	UCCGG CAUGC UCUCG<br>CCCGU GGACU CCCUG<br>GAGUC ACCCC AUGGC	N/A	N/A	0.868	0.647	0.827	0.841	m^6A site (Very high confidence)
11	7064	GGGCG GCCGG CUGGC<br>CUUUG AGACU GGCCC<br>ACCUC GUCUC UCCCA	N/A	N/A	0.865	0.598	0.772	0.814	m^6A site (Very high confidence)
12	7428	ACCCA GCAGG UGCAG<br>CCACA AAACU UACAG<br>AUGCA GCAGC AGAAC	N/A	N/A	0.833	0.483	0.776	0.792	m^6A site (Very high confidence)
13	8158	UUUUA UCUGG CCCCA<br>GGUAG AAACU UUUAU<br>CUAUU CUGAG AAAAC	N/A	N/A	0.854	0.375	0.784	0.802	m^6A site (Very high confidence)
14	8782	AUGUC AAACA UGAGA<br>UGUGU GGACU GUGGC<br>ACUUG CCUGG GUCAC	N/A	N/A	0.761	0.813	0.489	0.655	m^6A site (High confidence)

a-b. The efficiency of sgRNA were verified by qRT-PCR (a) and western blotting analysis (b). c. lentiCRISPR vectors containing sgRNAs (single-guide RNAs) targeting HNRNPC was designed by Beyotime. d. The prediction score distribution along the NOTCH1 sequence were shown by SRAMP (http://www.cuilab.cn/sramp). e. RIP-qRT-PCR for ACHN cells: showing the enrichment of m6A modification decrease in the region P2 in the MIR503HG overexpression. f. RIP-qRT-PCR for ACHN cells: detecting the enrichment of HNRNPC in MIR503HG KD in the NOTCH1-WT and NOTCH1-P2-MUT group. The statistical difference was assessed through 2-tailed Student's t- test in a, e-f. Error bars show the standard deviation (SD) from three independent experiments. **P* < 0.05; ***P* < 0.01; ****P* < 0.001.

Figure S7. Everolimus repressed LN metastasis via inducing MIR503HG.

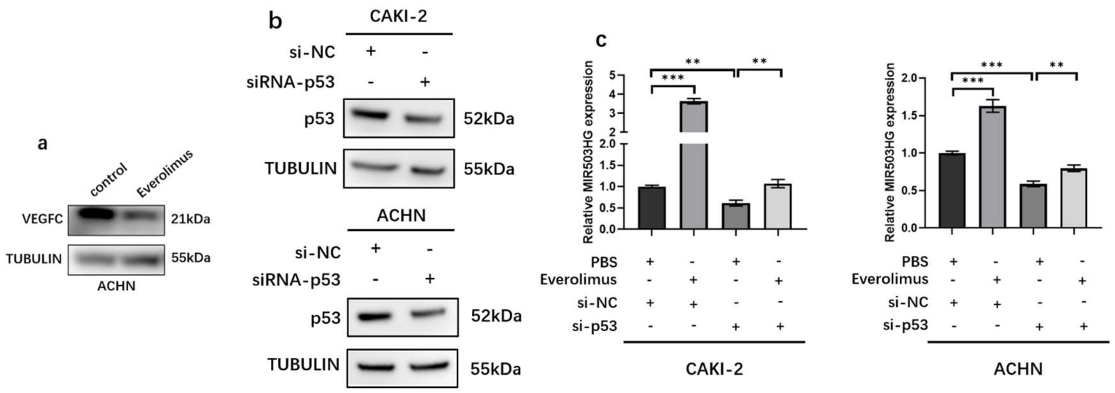

a. Western blotting to determine treatment effects of everolimus on VEGFC protein expression in ACHN cells. b. Western blot analysis for p53 protein levels with si-p53 in pRCC cells. c. MIR503HG expression was measured by Real-time PCR assays in pRCC cells expressing si-p53 or treated with Everolimus and si-p53 + Everolimus. Error bars show the standard deviation (SD) from at least three independent experiments. *P < 0.05; **P < 0.01.

Supplemental Table 1.Potential p53 binding site in MIR503HG promoter, predicted by JASPAR database.

Name	Score	Relative score	Start	End	Strand	Predicted sequence
MA0106.2.TP53	9.688334	0.83195	1090	1104	-	TCATGTCCTGAAATG
MA0106.2.TP53	7.958505	0.81093	850	864	+	ACTTGTGCTGAAATG
